# HPC restores mitochondrial homeostasis and ameliorates hypoxic stress through the GSK-3β/β-catenin signaling pathway

**DOI:** 10.1038/s41419-026-09080-7

**Published:** 2026-07-08

**Authors:** Wenxin Li, Qian Qu, Xingling Liu, Caixu Guo, Guo Shao, Baohui Ma, Jun Lv, Xiaoqiong Hao, Ruifang Qi

**Affiliations:** 1 Department of Basic and Forensic Medicine, Baotou Medical College, Baotou, PR China; 2 Institute of Neuroscience, Baotou Medical College, Baotou, PR China; 3 Beijing Key Laboratory of Hypoxic Conditioning Translational Medicine, Xuanwu Hospital, Capital Medical University, Beijing, PR China; 4 Center for Translational Medicine and Department of Laboratory Medicine, The Third People’s Hospital of Longgang District, Shenzhen, PR China

**Keywords:** Cellular neuroscience, Neuronal physiology, Organelles, Cell signalling

## Abstract

Hypoxic preconditioning (HPC) can increase the hypoxia tolerance of the mouse hippocampus both in vivo and in vitro by upregulating ATP levels, which may depend on mitochondrial homeostasis. The GSK-3β/β-catenin signaling pathway is involved in neuroprotection after brain ischemia. The aim of this study was to explore whether HPC can activate GSK-3β/β-catenin to improve the hypoxia tolerance of neuronal cells by enhancing mitochondrial homeostasis. In vitro and in vivo analyses revealed that HPC upregulates the activity of the GSK-3β/β-catenin pathway, maintaining mitochondrial morphological stability. Mechanistically, GSK-3β activity is negatively correlated with mitochondrial homeostasis. Pharmacological inhibition of GSK-3β reduced mitochondrial fission, whereas HPC suppressed GSK-3β expression to attenuate fission and preserve mitochondrial integrity. Conversely, GSK-3β overexpression abrogated HPC-mediated protection and exacerbated mitochondrial dysfunction. These findings elucidate a neuroprotective mechanism whereby HPC stabilizes mitochondrial dynamics via modulation of the GSK-3β/β-catenin pathway, providing novel experimental insights into HPC-mediated neuroprotection.

## Introduction

The tolerance of the brain, particularly the brainstem, hippocampus, and cortex, to ischemic/hypoxic insults is limited because of its high oxygen demand. Severe hypoxia induces irreversible neuronal damage through bioenergetic failure, mitochondrial dysfunction, inflammation, and apoptosis [1–3]. Ischemia/hypoxic preconditioning (I/HPC) is a procedure in which the body is given a sublethal hypoxic or ischemic stimulus to obtain a preadaptive response that is capable of attenuating or resisting damage caused by more severe ischemia and hypoxia [4]. Some studies have shown that HPC can exert neuroprotective effects by affecting mitochondrial homeostasis to achieve protection of the brain and nervous system [4, 5].

Mitochondria, which are dynamic organelles that govern energy production and apoptosis, maintain homeostasis through fusion–fission balance, mitophagy, and biogenesis [6–10]. Under hypoxia stress, cell differentiation, mitochondrial damage and nutrient deprivation, cells respond to activate mitophagy to remove dysfunctional or excessive mitochondria and maintain cell homeostasis [11–14]. Mitochondrial autophagy occurs under basal conditions (i.e., without induced mitochondrial damage) as opposed to stimulus-induced mitochondrial autophagy, and the molecular mechanisms by which the cell regulates mitochondrial homeostasis in the steady state are unclear [15, 16].

The GSK-3β/β-catenin pathway critically mediates postischaemic neuroprotection [17, 18], with evidence suggesting that HPC promotes neuroprotection through GSK-3β inactivation [19, 20]. However, whether this pathway enhances hippocampal hypoxia tolerance via the regulation of mitochondrial homeostasis remains unexplored. This study investigated how HPC modulates GSK-3β/β-catenin signaling to maintain mitochondrial homeostasis and improve neuronal survival under hypoxic stress. (Fig. S1)

## Materials and methods

### Cell culture and treatment

Mouse HT22 neuronal cell lines were obtained from Shanghai Yihe Applied Biotechnology Co., Ltd, and the company provides a cell identification report (Shanghai, China). Control: HT22 cells were cultured in DMEM (Cat. 12800-017; Gibco, USA) supplemented with 10% FBS (Cat. 11011-8611; Sijiqing, Hangzhou, China) under normoxic conditions. Hypoxia: HT22 cells were exposed to 1% O₂ and 5% CO₂ for 13 h, followed by 6 h of reoxygenation. The HPC group underwent four cycles of intermittent hypoxia (1–21% O₂ every 30 min) prior to 13 h of hypoxia and 6 h of reoxygenation, simulating preconditioning. All experiments were maintained in 5% CO₂ at 37 °C.

### Mouse model and grouping

Owing to limited animal availability and animal-welfare considerations, no formal a priori power analysis was performed. Only male mice were used in this study. Male ICR mice that were 6 weeks old and weighed 18–22 g were obtained from SPF (Beijing) Biotechnology Co., Ltd. The mice were given free access to tap water, fed a standard laboratory diet and kept in a controlled room with standard conditions (25 °C, 55% humidity, and 12 h light/dark cycle). All animal studies were performed according to procedures approved by the Ethics Review Committee for Animal Experimentation of Inner Mongolia University of Science and Technology, Baotou Medical College. The inclusion and exclusion criteria for animals/samples in this study were predetermined. Inclusion criteria: healthy animals within a specific range of age/weight. All mice used in the analysis were male ICR background, with random distribution within each experimental group.

### Animal ethics and study approval

All animal experiments were performed strictly in accordance with the national guidelines and institutional regulations for laboratory animal welfare. All animal experimental protocols were reviewed and approved by the Animal Ethics Committee of Baotou Medical College (Approval No: 202413). This study did not involve human participants; therefore, informed consent to participate was not applicable, and no identifiable human images are included in this manuscript.

### Neurobehavioural evaluation of mice

The climbing pole test and rotarod test were used to evaluate the motor balance ability, muscle grip strength and endurance of the mice. Climbing pole test: The mice were placed on a 50 cm high platform, and the time for the mice to reach the ground was recorded. Rotarod test: Prior to the experiment, the mice were trained on a rotating rod that gradually accelerated from 0 to 20 rpm. During the formal test, if a mouse lost its balance and fell onto the platform below, the rotation rod automatically stopped timing at the corresponding position. The time spent on the rod and the number of drops were recorded [21, 22].

### Nissl staining

The mice were perfused with 4% paraformaldehyde, after which their brains were collected. After sinking, the brain was transferred to 30% sucrose solution for dehydration and then cut into 30 μm slices using a frozen sectioning machine (CryoStar NX50; Thermo Fisher Scientific, US). The hippocampal region of the mouse brain slice was stained using a Nissl staining kit (Cat. G430; Solarbio, Beijing, China) according to the manufacturer’s instructions [23].

### Transmission electron microscopy

Mitochondrial ultrastructure in mouse hippocampal tissue was evaluated using transmission electron microscopy (TEM). Mouse hippocampal tissue was dissected and fixed in 2.5% glutaraldehyde for 2 h on ice and cut into 1–2 mm cubes. After being washed with 0.1 M phosphoric acid rinsing solution, the liver tissue was fixed in 1% osmium tetroxide for 1–2 h at 4 °C and then dehydrated and embedded in epoxy resin. The tissue was sliced into 70 nm sections using an ultramicrotome, mounted on copper grids, contrasted with 2% uranyl acetate and lead citrate, and observed with a transmission electron microscope. For analysis, the mitochondria of three animals in each group were traced.

### Immunofluorescence assay

After mouse hippocampal tissue and HT22 cell modeling were completed, the mouse brain tissue was fixed and dehydrated, frozen sections were prepared, and the HT22 cells were fixed with 4% paraformaldehyde after crawling to obtain the slices. The target antibody (GSK-3β: Cat. 12456S, CST, US; LC3: Cat. 4108, CST, US; and TOM20: Cat. 54316, SAB, China) was used according to the instructions. The next day, the sections were incubated with the corresponding fluorescent secondary antibody. The slices were sealed with an anti-quencher and photographed by laser confocal microscopy.

### Western blot analysis

Protein samples were prepared from mouse hippocampal tissue or cells. Afterward, the same concentration of protein samples was separated by SDS‒PAGE (8–15%). The corresponding antibodies against GSK-3β (Cat. 12456S; CST, US), p-GSK-3β (Cat. 558S; CST, US), β-catenin (Cat. 51067-2-AP; Proteintech, China), p-β-catenin (Cat. 28853-1-AP; Proteintech, China), TOM20 (Cat. 54316; SAB, China), TIM23 (Cat. 11123-1-AP; Proteintech, China), DRP1 (Cat. 40853; SAB, China), MFN2 (Cat. 56132; SAB, China), LC3B (Cat. T55992F; Abmart, Chain), NIX (Cat. 68118-1-lg; Proteintech, China), p-PINK1(Cat. 89010; CST, US), β-actin (Cat. sc-47778; SANTA, USA), histone (Cat. 4499S; CST, US), and β-tubulin (Cat. T6199; SIGMA, DEU) were added. The secondary antibodies included anti-rabbit IgG with anti-mouse IgG (1:12,000 dilution). The membranes were visualized using ECL (Cat. C1006A; Liweining, China) chemiluminescence reagents.

### CellTiter 96^®^ AQueous One Solution Cell Proliferation Assay (MTS)

HT22 cells were inoculated at a density of 1 × 10^3^ in 96-well plates. MTS (Cat. G358C; Promega, USA) solution was added to each well. The cells were incubated at 37 °C for 3 h, after which the optical density at 450 nm was measured using a microplate reader (Multiskan FC, Shanghai, China). The concentrations of the GSK-3β (Cat. HY-10182; MCE, USA) and β-catenin (Cat. HY-120697; MCE, USA) inhibitors used for the in vitro studies were determined with reference to the literature support provided by the company [24].

### Flow cytometry assessment of apoptosis

An Annexin V-APC/PI Apoptosis Detection Kit (KGA1030; KeyGen Biotech, China) was used to detect apoptosis according to the instructions. In brief, the HT22 cells were collected and then resuspended in binding buffer. After that, 5 μL of Annexin V-APC and 5 μL of PI were added to the cells, and they were allowed to react at 25 °C for 15 min in the dark. The number of apoptotic cells was determined by flow cytometry.

### Cell Insight CX7 HCS platform assay

Mitochondria were observed on the Cell Insight CX7 HCS platform (Thermo Fisher Scientific, USA) at the core facility Key Laboratory of Hypoxic Neuroprotection by using the fluorescent dyes Hoechst (which stains all the cell nuclei; signal registration at 386 nm) and MitoTracker^TM^ Red CMXRos (Cat. M7512; Invitrogen, US; which stains the mitochondria; signal registration at 549 nm).

### MitoTracker Red CMXRos probe staining

HT22 cells were inoculated into confocal dishes for walling. The prepared MitoTracker Red CMXRos (Cat. C035-50 µg, Beyotime, China) probe working solution with Hoechst reagent was added, and the cells were incubated together for 15 min in a 37 °C incubator. The medium was replaced with fresh medium. Pictures were taken under a laser microscope, and the experiment was repeated three times (signal registration at 579–599 nm).

### Mitochondrial membrane potential (MMP) assay

HT22 cells were inoculated in confocal dishes. JC-1 staining (Cat. BL71A; Biosharp, China) working solution was added and mixed, and the cells were incubated in an incubator at 37 °C for 20 min. The medium was replaced with fresh medium. Pictures were taken under a laser microscope, and the experiment was repeated three times (JC-1 monomer: signal registration at 515–529 nm; JC-1 polymer: signal registration at 585–590 nm).

### Transfection of HT22 cells

HT22 cells were transfected with the GSK-3β, LC3 and β-catenin overexpression plasmids using M5 HiPer “Transbid” Lipo2000 Transfection Reagent (MF-135-01; PolyMed, Beijing, China). Transfected cells were obtained for subsequent Western blot or immunofluorescence analysis.

### Statistical analysis

GraphPad Prism 8 software was used to perform *t*-tests for all the statistical analyses. *p* < 0.05 indicated a statistically significant difference, and all the values are the mean ± SEM. Multiple-group comparisons were performed by one-way analysis of variance (ANOVA), whereas two-group differences were evaluated by Student’s *t*-tests. Each experiment was repeated at least three times.

## Results

### HPC attenuates hypoxia-induced neuronal damage in the mouse hippocampus, enhances motor balance function and exerts neuroprotective effects in mice

**Fig. 1:**
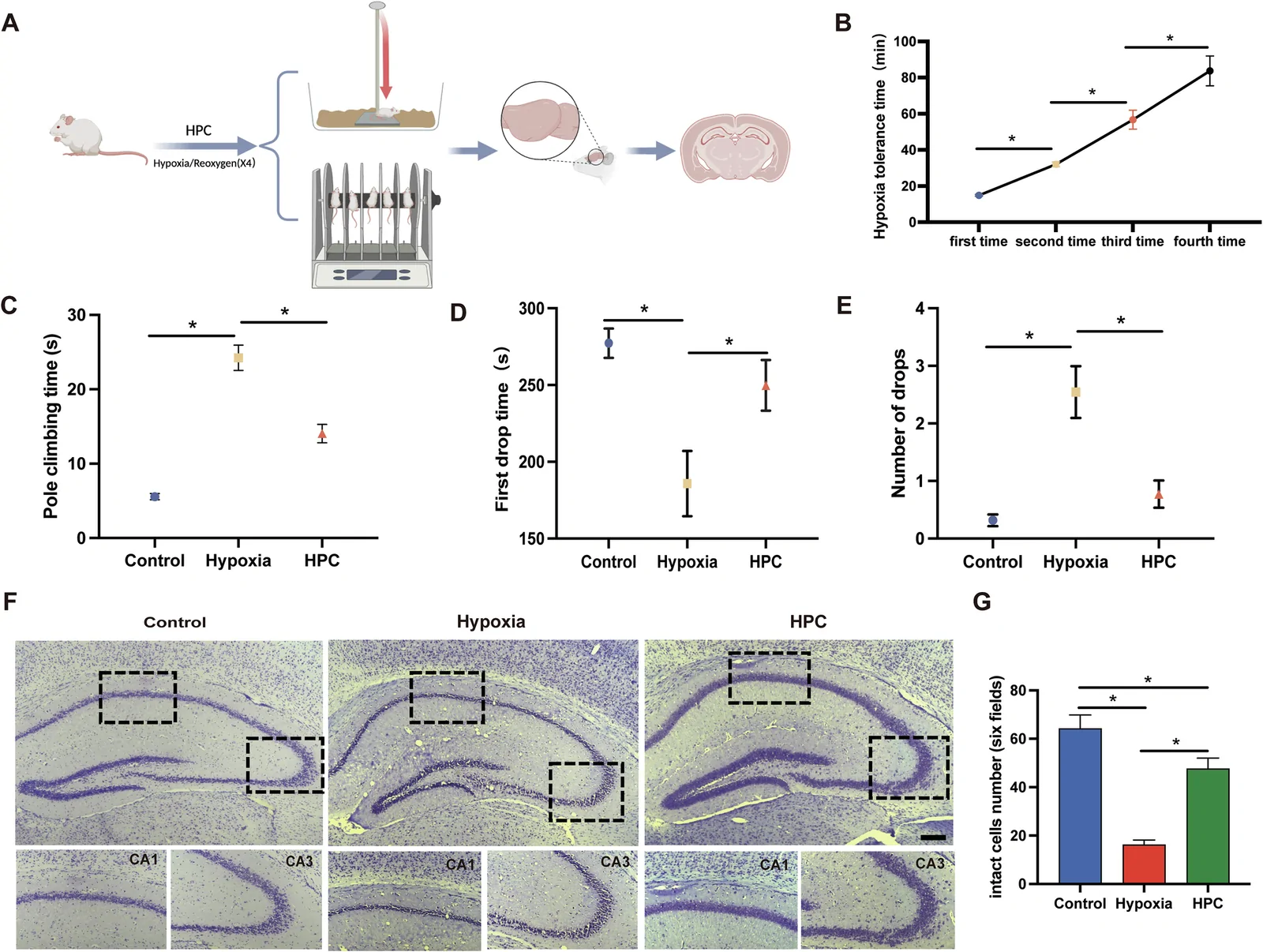
Effects of HPC on motor function and mouse hippocampal nerve cells in mice. **A** Schematic diagram of mouse modeling and neurobehavioral evaluation; **B** four hypoxia tolerance times in mice (*n* = 6); **C** fall time of the mouse climbing the pole; **D** time to first drop in the mouse baton twirling experiment; **E** number of drops in the mouse baton twirling experiment; **F** Nissl staining of the mouse hippocampus; scale bar = 100 μm (*n* = 6). **G** The number of Nissl-positive cells (*n* = 3). When the mouse model was successfully established, and samples were obtained on the same day, the samples were classified as the D0 group. Samples of tissue obtained after 1 day were classified as group D1, and samples obtained after 2 days were classified as group D2. GraphPad Prism 8 software was used to perform *t*-tests for all statistical analyses. All the values are the mean ± SEM. *p < 0.05 indicates a statistically significant difference.

To investigate the protective effect of HPC on the nervous system of mice, we treated mice with HPC modeling and assessed the statistics by neurobehavioral evaluation and morphological observation of mouse hippocampal nerve cells (Fig. 1A). The results revealed that HPC significantly prolonged the hypoxia tolerance time (*p* < 0.05; *n* = 6; Fig. 1B), significantly increased the speed of pole climbing, improved the muscle strength, prolonged the stay time on the rotating rod, and significantly enhanced limb coordination, with the differences in the data exhibiting statistical significance (*p* < 0.05; *n* = 6; Fig. 1C–E). The morphology revealed that mouse hippocampal neurons in the HPC group had reduced vacuole-like degeneration, the proportions of nidus granules and normal cells were greater than those in the hypoxia group, and regular arrangement was restored. The number of Nissl-positive cells in the hypoxia group was significantly lower than that in the control group, and the number in the HPC group was significantly greater than that in the hypoxia group (Fig. 1F, G). These findings suggest that HPC alleviates hypoxia-induced motor dysfunction and enhances neuroprotection by maintaining neuronal structural integrity.

### HPC attenuates mitochondrial fission and promotes mitochondrial fusion in the hippocampal neurons of mice, thus exerting a neuroprotective effect

**Fig. 2:**
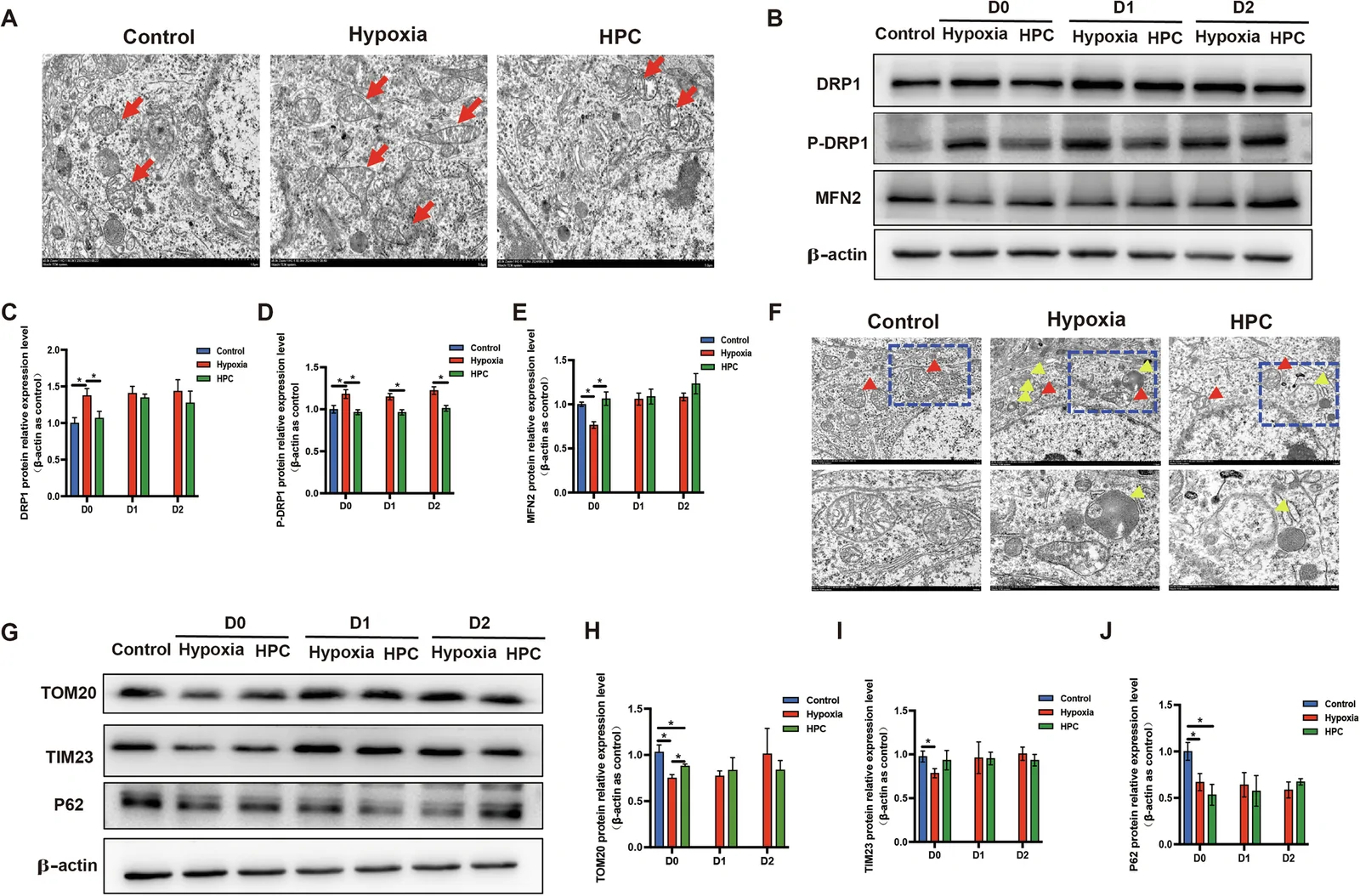
Effects of HPC on mitochondrial homeostasis in neuronal cells of the mouse hippocampus. **A** Morphology of mitochondrial division by transmission electron microscopy (*n* = 3); **B** representative protein band of mitochondrial fission and fusion proteins; **C** quantification of DRP1 (*n* = 6); **D** quantification of p-DRP1 (*n* = 6); **E** quantification of MFN2 (*n* = 6); **F** number of autophagic lysosomes: red for mitochondria and yellow for autophagosomes, *n* = 3; **G** representative protein band of mitochondrial membrane proteins with mitochondrial autophagy proteins; **H** quantification of TIM23 (*n* = 6); **I** quantification of TOM20 (*n* = 6); **J** quantification of P62 (*n* = 6). GraphPad Prism 8 software was used to perform *t*-tests for all the statistical analyses. All the values are the mean ± SEM. *p < 0.05 indicates a statistically significant difference.

Next, whether the protective effect of HPC on hippocampal neurons occurred through the protection of mitochondrial homeostasis was verified. TEM revealed elongated mitochondria with disorganized cristae in hypoxic hippocampal neurons, whereas HPC mitigated fission and restored their ultrastructure (Fig. 2A). Hypoxia upregulated the expression of the fission protein DRP1/p-DRP1 and suppressed the expression of the fusion protein MFN2, whereas HPC reversed these effects (*p* < 0.05; *n* = 6; Fig. 2B–E).

TOM20 and TIM23 proteins are the inner and outer membrane proteins of mitochondria, respectively. When mitochondrial autophagy occurs, the protein levels of TOM20 and TIM23 decrease. The process of mitophagy involves the formation of early autophagosomes and the hydrolysis of autolysosomes in the later stage. Therefore, we detected changes in TOM20 and TIM23 protein levels and observed changes in mitophagy by TEM. Reduced TOM20 and TIM23 levels and increased numbers of autophagosomes under hypoxia. Compared with those in the hypoxia group, the number of autophagosomes and the degradation of membrane proteins in the HPC group were attenuated but were still greater than those in the control group. In addition, compared with those in the control group, P62 protein levels in both groups were significantly lower, and the data were statistically significant (*p* < 0.05; *n* = 6; Fig. 2F–J). In conclusion, HPC regulates fusion and autophagy by inhibiting mitochondrial division and maintaining mitochondrial homeostasis under hypoxic stress in a balanced manner.

### HPC attenuates mitochondrial fragmentation and increases cell survival in HT22 cells

**Fig. 3:**
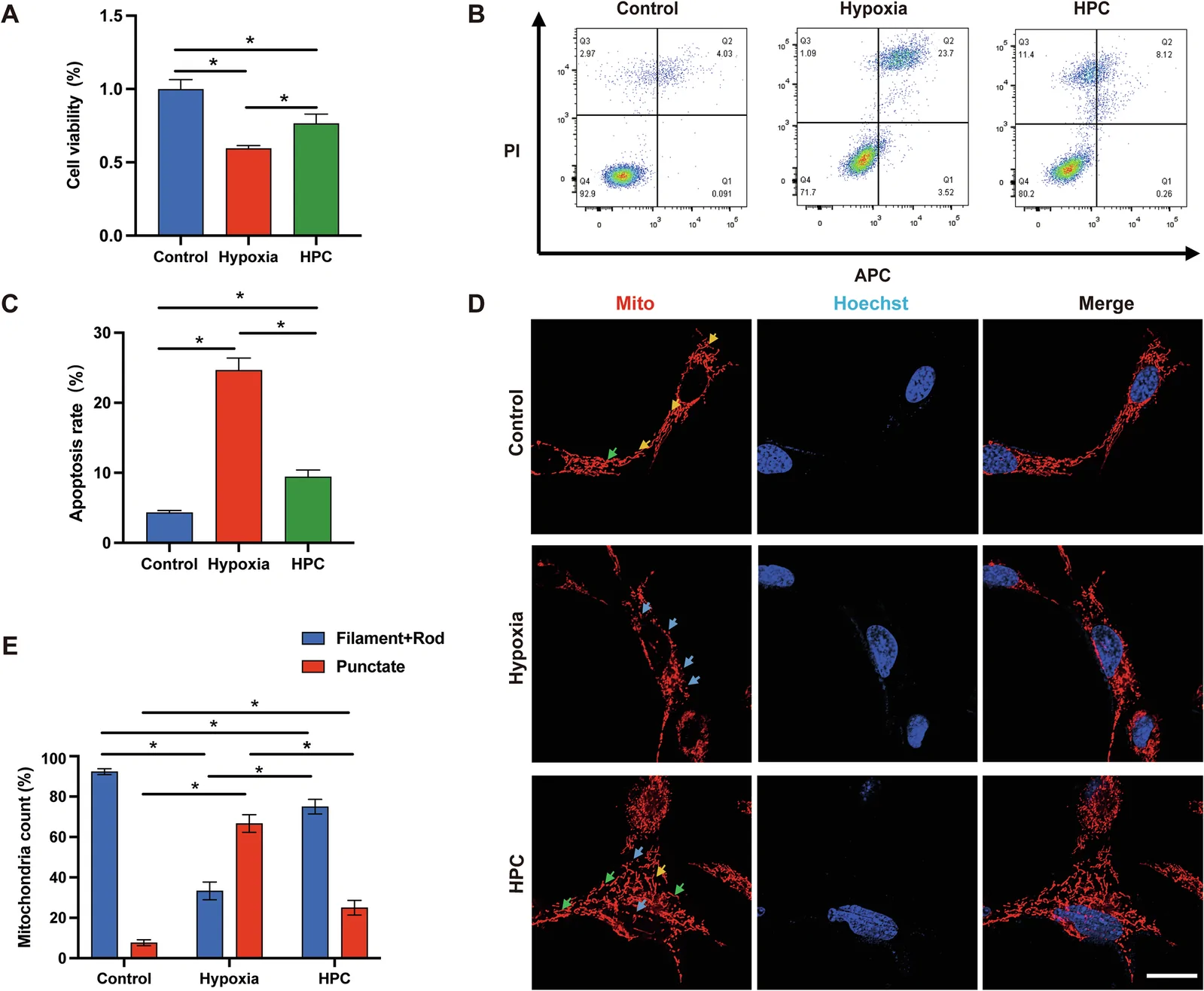
Effect of HPC on HT22 cell viability and mitochondria. **A**. Statistical graph of cell viability (*n* = 3); **B** cell apoptosis was detected by flow cytometry; **C** quantification of the apoptosis rate (*n* = 3); **D** representative images of mitochondrial morphology labeled with MitoTracker Red; the yellow arrows indicate mitochondrial filaments, the green arrows indicate short tubular mitochondria, and the blue arrows indicate fragments of mitochondria. scale bar = 20 μm; **E** the ratio of different mitochondrial morphologies (*n* = 3). GraphPad Prism 8 software was used to perform *t*-tests for all the statistical analyses. All the values are the mean ± SEM. *p < 0.05 indicates a statistically significant difference.

The protective effect of HPC on HT22 cells was explored ex vivo. The results revealed a significant decrease in HT22 cell viability in the hypoxia group. Compared with that in the hypoxia group, the viability of HT22 cells in the HPC group was significantly greater, but that in the control group was lower. Flow cytometry revealed the same trend, and the data were statistically significant, suggesting successful modeling (*p* < 0.05; *n* = 3; Fig. 3A–C).

Observations of mitochondrial morphology revealed that the mitochondria were tightly packed around the nucleus in the control group and that the point-like distribution was fragmented in the hypoxia group, whereas the number of mitochondrial rod-like structures increased and fragmentation decreased in the HPC group (*p* < 0.05; *n* = 3; Fig. 3D, E). These results suggest that HPC can increase cell viability and maintain normal mitochondrial morphology.

### HPC attenuates mitochondrial fission, promotes mitochondrial fusion, and exerts a protective effect on neuronal cells

**Fig. 4:**
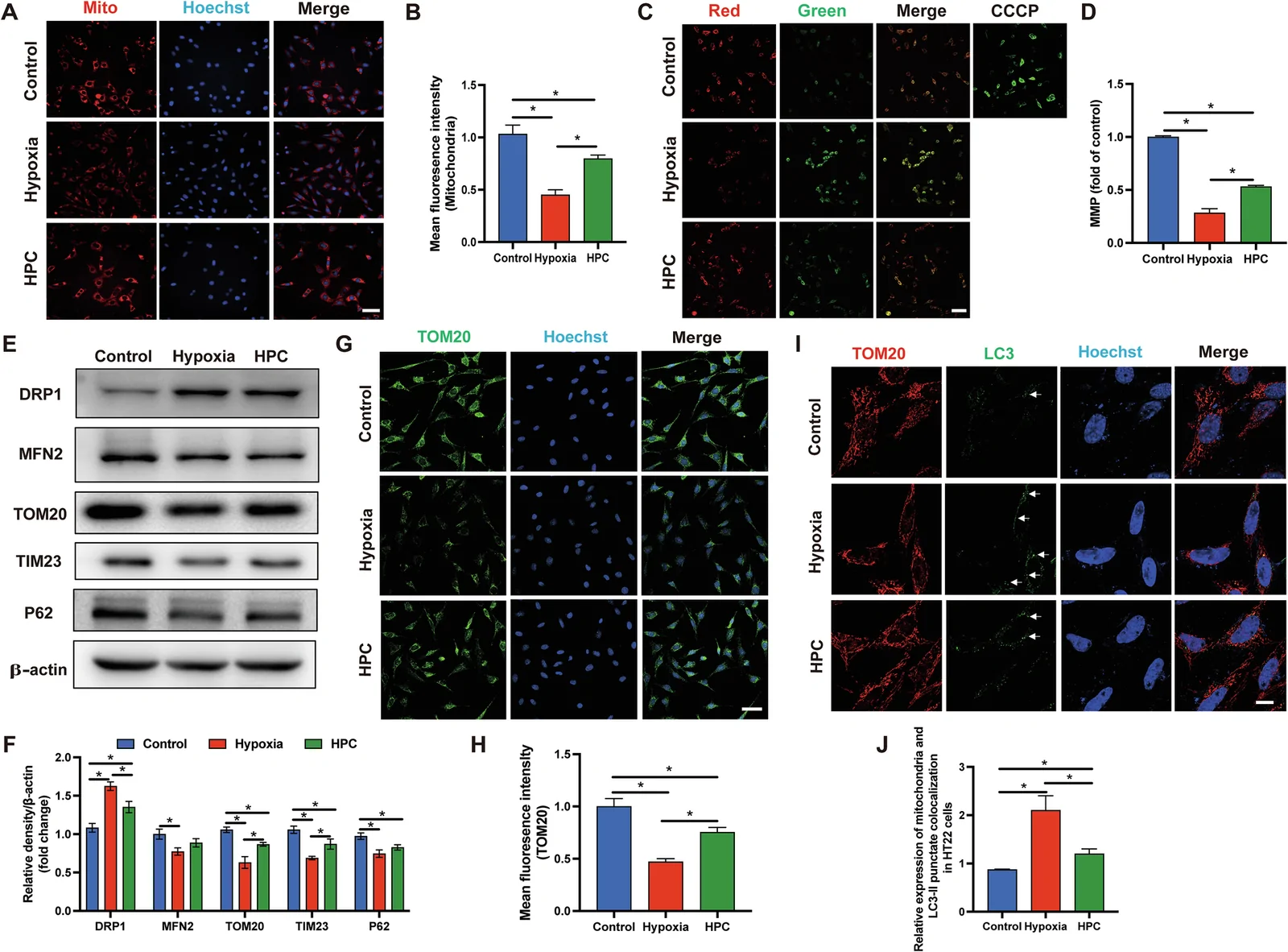
Effects of HPC on HT22 cell viability, mitochondrial membrane potential and cellular mitochondrial homeostasis. **A** Assessment of MMP changes using MitoTracker^TM^ Red CMXRos and the Cell Insight CX7 HCS platform, scale bar = 50 μm (*n* = 3); **B** statistical analysis of the mitochondrial fluorescence intensity; **C** assessment of MMP changes using JC-1 staining, scale bar = 50 μm; **D** quantification of MMP (*n* = 3); **E** representative protein band of mitochondrial fission and autophagy protein; **F** quantification of protein (*n* = 6); **G** TOM20 immunofluorescence assay, scale bar = 50 μm; **H** TOM20 immunofluorescence intensity statistics (*n* = 3); **I** Schematic diagram of TOM20 costaining with LC3, scale bar = 20 μm. **J** The proportion of LC3 coincident with TOM20 (*n* = 3). GraphPad Prism 8 software was used to perform *t*-tests for all the statistical analyses. All the values are the mean ± SEM. *p < 0.05 indicates a statistically significant difference.

To verify whether HPC attenuates mitochondrial division, activates autophagy and maintains mitochondrial homeostasis in HT22 cells to play a protective role, we performed CellInsight CX7 HCS platform and MMP assays on the cells. After hypoxia treatment, the MMP was reduced, whereas HPC was able to restore MMP levels (*p* < 0.05; *n* = 3; Fig. 4A–C). Western blot results revealed that the mitochondrial split protein DRP1 was upregulated and that the expression of the fusion protein MFN2 and mitochondrial marker proteins TOM20 and TIM23 decreased in the hypoxia group, whereas the opposite trend was observed in the HPC treatment group. Moreover, the P62 protein level significantly decreased in the HPC and hypoxia groups, indicating that both autophagy pathways were activated and that the difference was statistically significant (*p* < 0.05; *n* = 6; Fig. 4D, E).

Immunofluorescence staining revealed that the TOM20 immunofluorescence intensity was consistent with the change in the protein level, and the difference in the data was statistically significant (*p* < 0.05; *n* = 3; Fig. 4F, G). LC3 fluorescence revealed that the recombination rate of HPC-treated LC3 with TOM20 was significantly greater than that of the hypoxia group (*p* < 0.05; *n* = 3; Fig. 4H, I). To further determine the effect of HPC on mitophagic flux, we performed a flux assay with chloroquine (CQ) treatment. After CQ treatment followed by model induction, a pronounced accumulation of LC3II was observed in the hypoxia group, indicating that hypoxia alone induces a strong autophagic flux. Following the blockade of autophagic degradation by CQ, the HPC group exhibited extensive formation of tight “autophagosome–mitochondria–lysosome” complexes. This directly demonstrates that HPC induces highly selective mitophagy, in which autophagosomes precisely encapsulate mitochondria (Tom20-positive) and successfully deliver them to lysosomes. Moreover, Pearson’s correlation coefficient revealed significantly higher LC3/TOM20 co-localization in the HPC group than in the hypoxia group. (*p* < 0.05; *n* = 3; Fig. S4A, B).

These results indicated that HPC maintained mitochondrial homeostasis by inhibiting DRP1-mediated mitochondrial division, promoting fusion protein expression and activating autophagy, thus exerting neuroprotective effects; however, the specific underlying molecular mechanisms still need to be explored in depth.

### HPC activates the GSK-3β/β-catenin signaling pathway in the mouse hippocampus

To investigate whether the activity of the GSK-3β/β-catenin signaling pathway is involved in HPC-mediated neuroprotection, we assessed the protein levels of its key regulators. Western blot analysis was used to measure the expression of total GSK-3β, p-GSK-3β (Ser9), total β-catenin, and p-β-catenin (Ser675). Additionally, immunofluorescence staining was performed to visualize the distribution and expression of GSK-3β in cells.

**Fig. 5:**
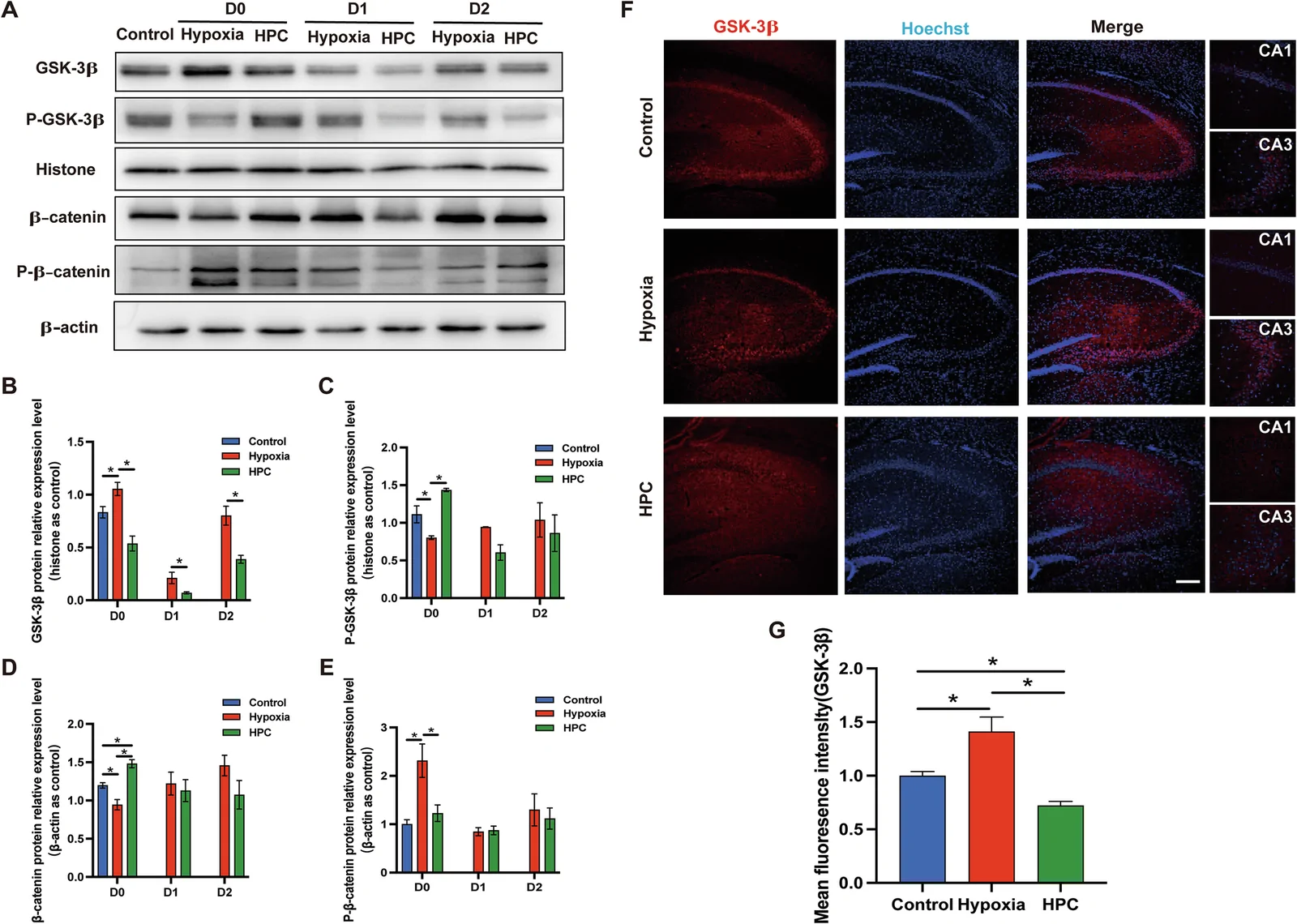
Effect of HPC on the GSK-3β/β-catenin signaling pathway in the mouse hippocampus. **A**. Representative protein band of the GSK-3β/β-catenin signaling pathway; **B** quantification of GSK-3β (*n* = 6); **C** quantification of p-GSK-3β (*n* = 6); **D** quantification of β-catenin (*n* = 6); **E** quantification of p-β-catenin (*n* = 6); **F** schematic representation of GSK-3β immunofluorescence in the mouse hippocampal region, scale bar = 200 μm; **G** statistical graph of GSK-3β immunofluorescence intensity (*n* = 6). GraphPad Prism 8 software was used to perform *t*-tests for all the statistical analyses. All the values are the mean ± SEM. *p < 0.05 indicates a statistically significant difference.

When the mouse model was successfully established, and samples were obtained on the same day, the samples were classified as the D0 group. Samples of tissue obtained after 1 day were classified as group D1, and samples obtained after 2 days were classified as group D2. In the D0 group, Western blot results showed that, compared with the control group, the hypoxia group presented increased GSK-3β and p-β-catenin protein levels and decreased β-catenin and p-GSK-3β protein levels. HPC treatment significantly decreased GSK-3β expression and upregulated β-catenin expression, and the differences in the data were statistically significant (*p* < 0.05, *n* = 6, Fig. 5A–E). This pattern, however, was not sustained at D1 and D2. Immunofluorescence staining revealed that the changes in the immunofluorescence intensity of GSK-3β were consistent with the protein levels, and the differences in the data were statistically significant (*p* < 0.05; *n* = 3; Fig. 5F, G). These results suggest that HPC may activate the GSK-3β/β-catenin signaling pathway in the hippocampus of mice during early hypoxic preconditioning (D0). However, over time, its role may be replaced by other regulatory mechanisms. The specific regulatory mechanisms that affect the GSK-3β/β-catenin pathway during late hypoxic preconditioning remain unclear.

### HPC activates the GSK-3β/β-catenin signaling pathway in HT22 cells

**Fig. 6:**
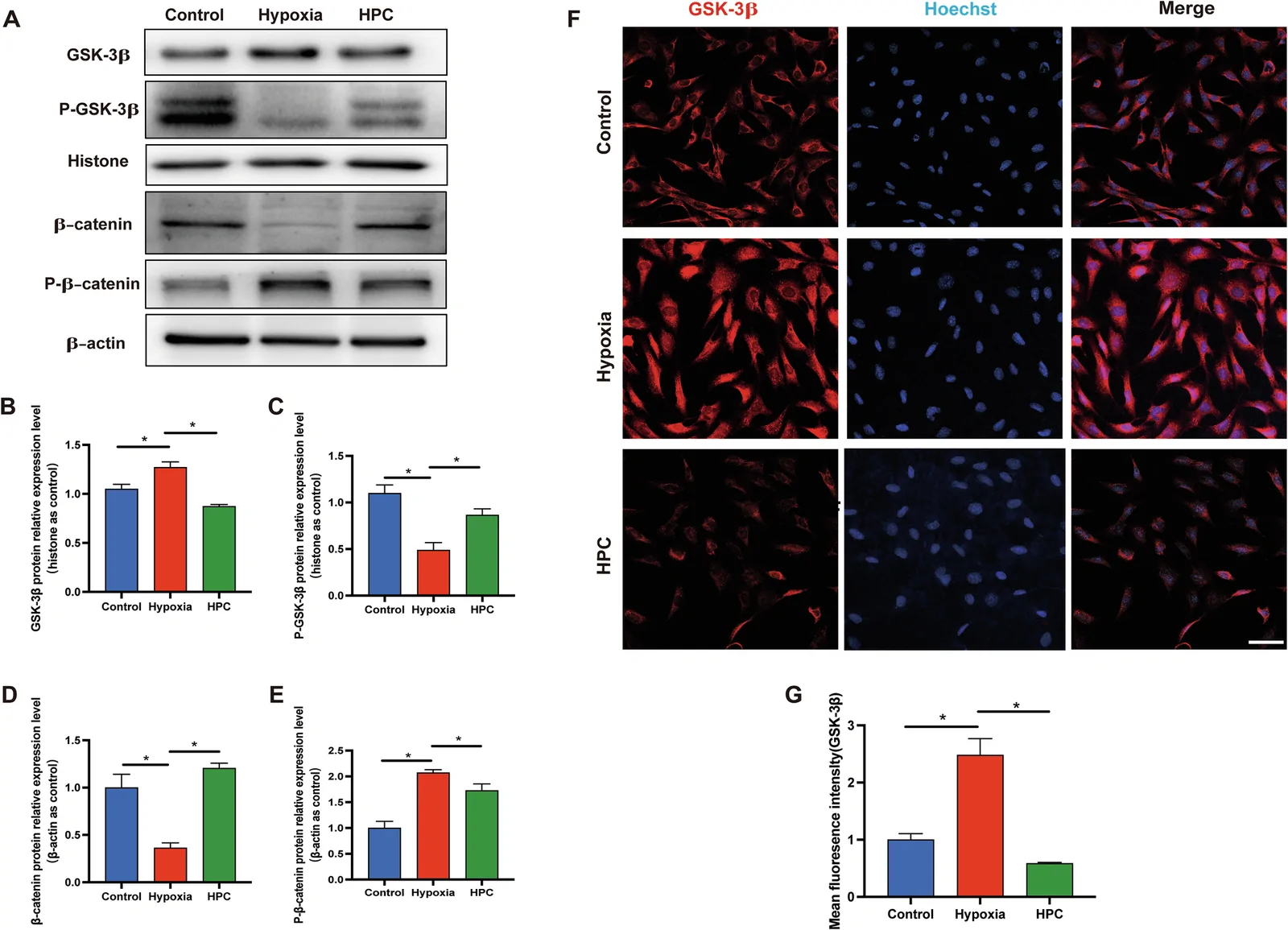
Effect of HPC on the activity of the GSK-3β/β-catenin signaling pathway in HT22 cells. **A** Representative protein band of the GSK-3β/β-catenin signaling pathway; **B** quantification of GSK-3β (*n* = 6); **C** quantification of p-GSK-3β (*n* = 6); **D** quantification of β-catenin (*n* = 6); **E** quantification of p-β-catenin (*n* = 6); **F** schematic diagram of immunofluorescence of HT22 cell GSK-3β, scale bar = 50 μm; **G** statistical graph of the GSK-3β immunofluorescence intensity (*n* = 6). GraphPad Prism 8 software was used to perform *t*-tests for all the statistical analyses. All the values are the mean ± SEM. *p < 0.05 indicates a statistically significant difference.

The effect of HPC on the GSK-3β/β-catenin signaling pathway was detected at the in vitro level by Western blotting. Compared with those in the control group, the GSK-3β and p-β-catenin protein levels in the hypoxia group were increased, and the p-GSK-3β protein level was decreased. Compared with those in the hypoxia group, the GSK-3β and p-β-catenin protein levels in the HPC group decreased, and the p-GSK-3β protein level increased, which was consistent with the results of in vivo experiments in mice, and the differences in the data were statistically significant (*p* < 0.05; *n* = 6; Fig. 6A–D).

Immunofluorescence staining revealed that the changes in the immunofluorescence intensity of GSK-3β were consistent with those in the protein levels, and the differences in the data were statistically significant (*p* < 0.05; *n* = 3; Fig. 6E, F). The above in vitro and in vivo results suggest that the GSK-3β/β-catenin pathway may be involved in HPC-mediated hypoxic neuroprotection, but whether HPC exerts a protective effect by affecting mitochondrial homeostasis is not clear.

### The inhibition of GSK-3β expression, activation of the GSK-3β/β-catenin pathway, and enhancement of mitochondrial homeostasis are involved in HPC-mediated neuroprotection

**Fig. 7:**
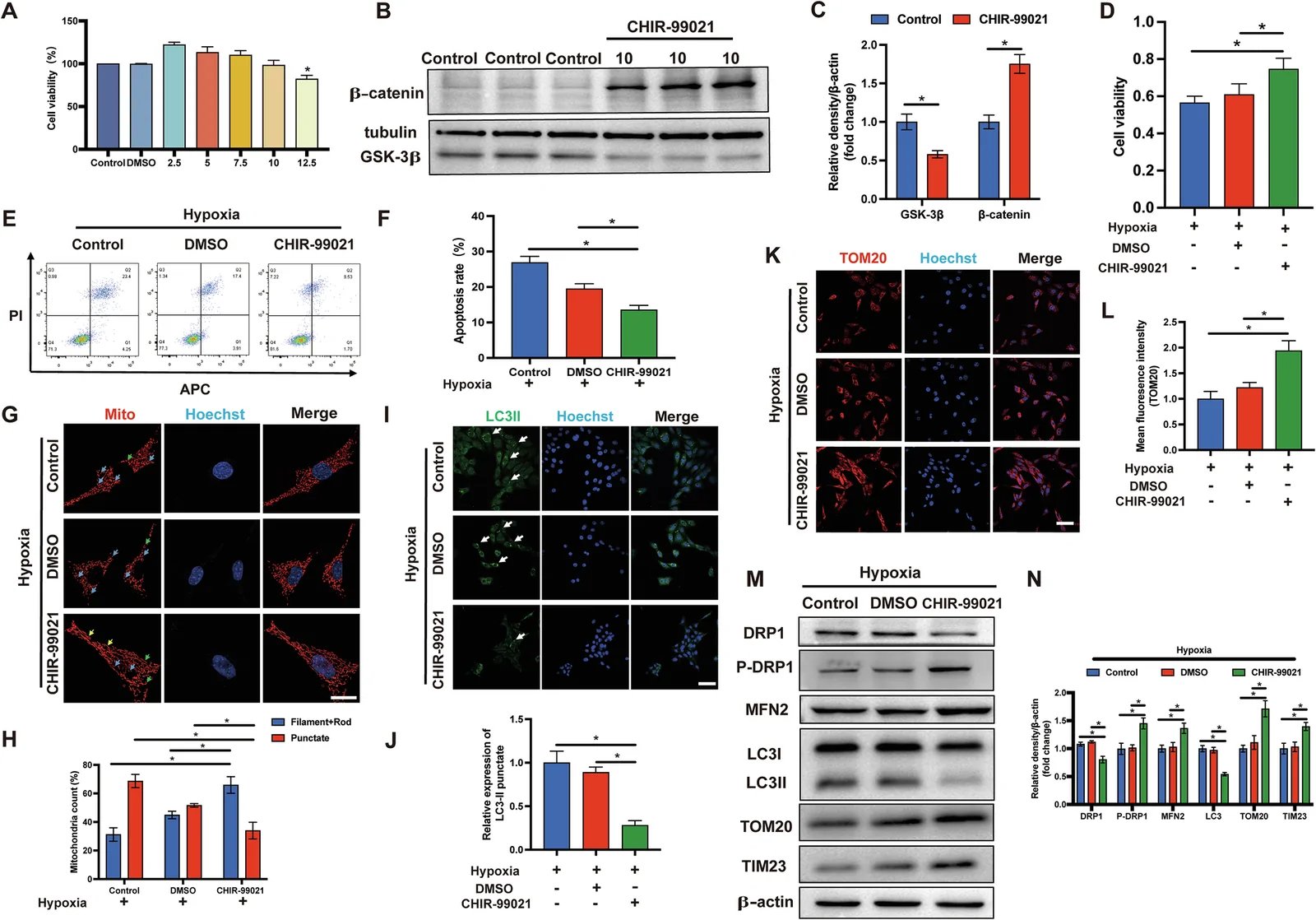
Effects of the inhibition of GSK-3β expression on mitochondrial homeostasis and HPC-mediated neuroprotection. **A** Statistical chart of cell viability (*n* = 3); **B** representative protein band of the GSK-3β inhibitor validation protein; **C** quantification of the activity of the GSK-3β/β-catenin signaling pathway (*n* = 6); **D** statistical plot of the viability of the HT22 cells after the addition of CHIR99021 (*n* = 3); **E** cell apoptosis was detected by flow cytometry; **F** quantification of the apoptosis rate (*n* = 3); **G** representative images of mitochondrial morphology labeled with MitoTracker Red; the yellow arrows indicate mitochondrial filaments, the green arrows indicate short tubular mitochondria, and the blue arrows indicate fragments of mitochondria; scale bar = 20 μm; **H** the ratio of different mitochondrial morphologies (*n* = 3). **I** LC3II immunofluorescence, scale bar = 50 μm; **J** statistical graph of the LC3 immunofluorescence intensity (*n* = 3); **K** TOM20 immunofluorescence, scale bar = 50 μm; **L** statistical graph of the TOM20 immunofluorescence intensity (*n* = 3); **M** representative protein bands of mitochondrial homeostasis-associated proteins; **N** quantification of mitochondrial homeostasis-associated proteins (*n* = 3). GraphPad Prism 8 software was used to perform *t*-tests for all the statistical analyses. All the values are the mean ± SEM. *p < 0.05 indicates a statistically significant difference. HT22 cells treated with the solvent control were defined as the DMSO group, and HT22 cells treated with CHIR99021 were defined as the CHIR99021 group.

The present study investigated the role of GSK-3β in hypoxic neuroprotection through the regulation of mitochondrial homeostasis. Experimental screening revealed that the GSK-3β inhibitor CHIR99021 (10 µM) significantly reduced the GSK-3β protein level and activated the GSK-3β/β-catenin pathway (*p* < 0.05; *n* = 6; Fig. 7A–C). Under hypoxic conditions, cell viability was elevated, apoptosis was reduced in the inhibitor-treated group (*p* < 0.05; *n* = 3; Fig. 7D–F), and the mitochondrial morphology was more intact (*p* < 0.05; *n* = 3; Fig. 7G, H). Immunofluorescence staining for LC3 and TOM20 revealed that the number of autophagosomes was significantly lower in the group treated with the inhibitor and hypoxia than in the group treated with hypoxia alone, and the intensity of TOM20 immunofluorescence was significantly greater than that in the group treated with hypoxia alone, with statistically significant differences observed in the data. Meanwhile, LC3 co-localization with TOM20 showed more complete mitochondrial morphology (*p* < 0.05; *n* = 3; Figs. 7I–L and S2G).

Western blot results revealed that in the inhibitor-treated group under hypoxic conditions, the DRP1 protein level was reduced, the MFN2 protein level was increased, the expression of the autophagy marker LC3II/I was reduced, the expression of the mitochondria-associated proteins TOM20/TIM23 was increased, and the p-PINK1 and NIX were reduced (*p* < 0.05; *n* = 6; Figs. 7M, N and S2D, E). These results suggest that inhibition of GSK-3β can alleviate hypoxia-induced neuronal cell injury by regulating mitochondrial dynamic homeostasis.

### Overexpression of GSK-3β, inhibition of the GSK-3β/β-catenin pathway, reduction in mitochondrial homeostasis and attenuation of HPC-regulated neuroprotection

**Fig. 8:**
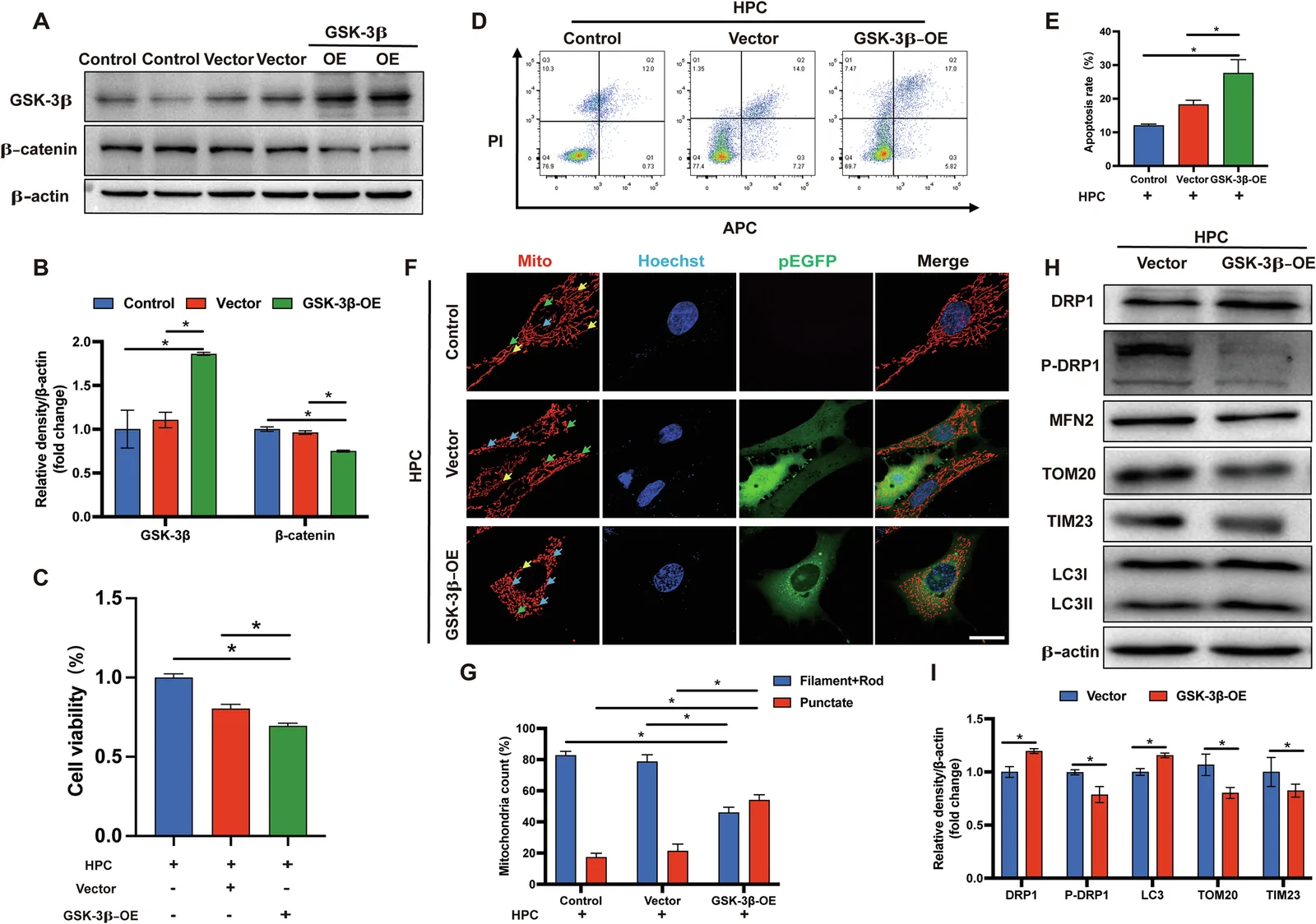
Effects of the overexpression of GSK-3β on mitochondrial homeostasis and HPC-mediated neuroprotection. **A** Representative protein bands of GSK-3β overexpression validation protein; **B** quantification of the activity of the GSK-3β/β-catenin signaling pathway (*n* = 6); **C** statistical plot of cell viability after modeling treatment in HT22 cells overexpressing GSK-3β (*n* = 3); **D** cell apoptosis was detected by flow cytometry; **E** quantification of the apoptosis rate (*n* = 3); **F** representative images of mitochondrial morphology labeled with MitoTracker Red; the yellow arrows indicate mitochondrial filaments, the green arrows indicate short tubular mitochondria, and the blue arrows indicate fragments of mitochondria; scale bar = 20 μm; **G** the ratio of different mitochondrial morphologies (*n* = 3); **H** representative protein bands of mitochondrial homeostasis-associated proteins; **I** quantification of mitochondrial homeostasis-associated proteins (*n* = 6). GraphPad Prism 8 software was used to perform t-tests for all the statistical analyses. All the values are the mean ± SEM. *p < 0.05 indicates a statistically significant difference. HT22 cells transfected with an empty plasmid were defined as the vector group, and HT22 cells transfected with the GSK-3β plasmid were defined as the GSK-3β-OE group.

The overexpression of GSK-3β in HT22 cells significantly increased the expression of GSK-3β, decreased the expression of β-catenin and inhibited the activity of the GSK-3β/β-catenin pathway, with statistically significant differences in the data (*p* < 0.05; *n* = 6; Figs. 8A–B and S2A). After HPC treatment, cell viability decreased, and apoptosis increased in the overexpression group (*p* < 0.05; *n* = 3; Fig. 8C–E), and mitochondrial morphology was more fragmented (*p* < 0.05; *n* = 3; Figs. 8F, G and S2F).

Western blot results revealed that after HPC treatment, compared with cells in the vector group, cells in the overexpression group presented increased levels of the DRP1 protein and decreased levels of the MFN2 protein, suggesting that the overexpression of GSK-3β enhances mitochondrial division. Moreover, TOM20 and TIM23 protein levels were reduced, LC3II/I protein levels were increased, and the p-PINK1 and NIX were reduced (*p* < 0.05; *n* = 6; Figs. 8H, I and S2B, C). These findings suggest that GSK-3β overexpression exacerbates mitochondrial dysfunction and impairs the protective effect of HPC.

### β-catenin is involved in the regulation of mitochondrial homeostasis and exerts neuroprotective effects

**Fig. 9:**
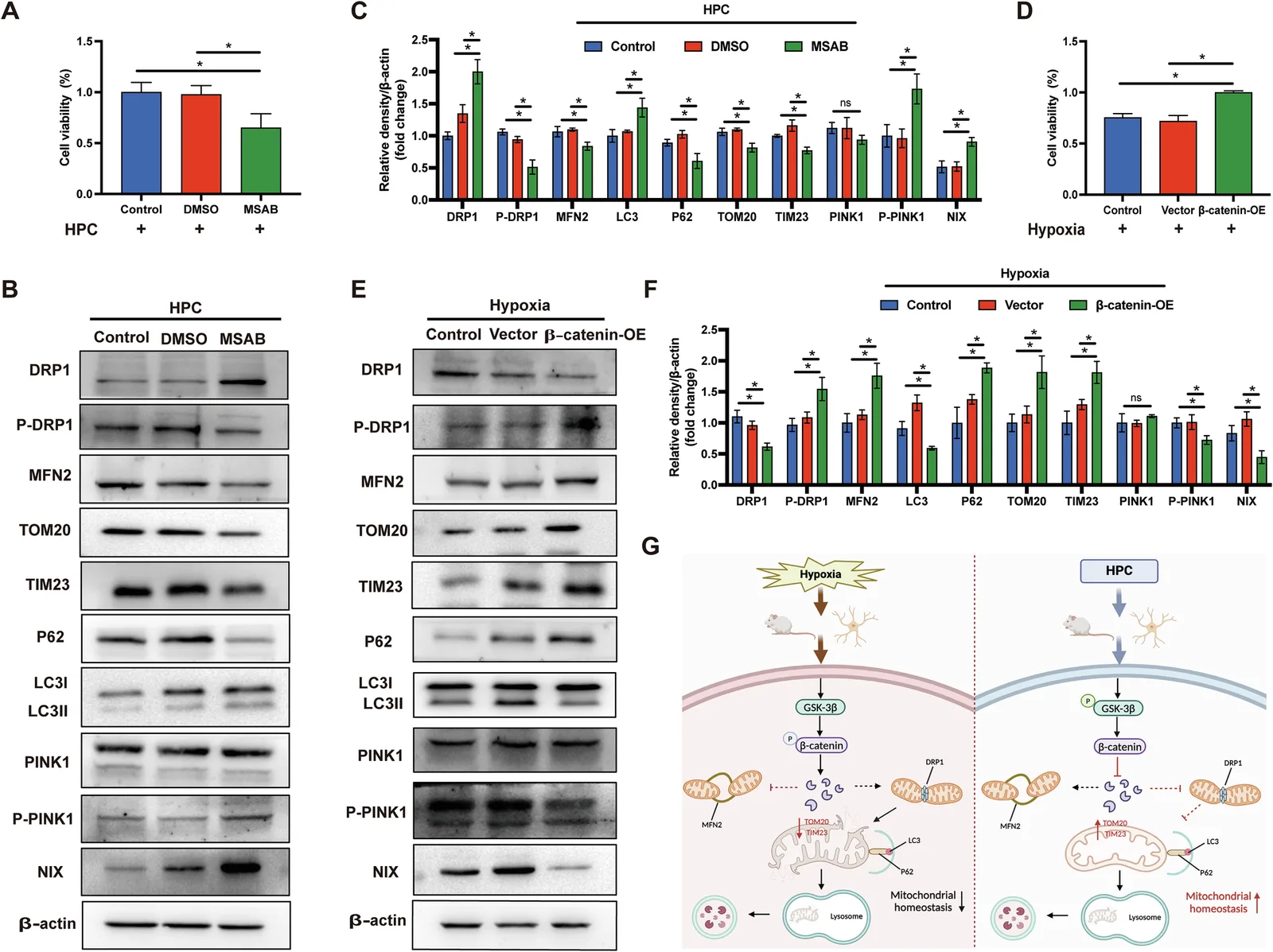
Effects of the inhibition and overexpression of β-catenin on mitochondrial homeostasis and HPC-mediated neuroprotection. **A** Statistical plot of the viability of HT22 cells treated with modeling after the addition of MSAB (*n* = 3); **B** representative protein bands of mitochondrial homeostasis-associated proteins; **C** quantification of mitochondrial homeostasis-associated proteins (*n* = 6); **D** statistical plot of cell viability after modeling treatment of HT22 cells overexpressing β-catenin; **E** representative protein bands of mitochondrial homeostasis-associated proteins; **F** quantification of mitochondrial homeostasis-associated proteins (*n* = 6); **G** mechanistic diagram of the GSK-3β/β-catenin signaling pathway. GraphPad Prism 8 software was used to perform *t*-tests for all the statistical analyses. All the values are the mean ± SEM. *p < 0.05 indicates a statistically significant difference. HT22 cells transfected with an empty plasmid were defined as the vector group, and HT22 cells transfected with β-catenin plasmid were defined as the β-catenin-OE group. HT22 cells treated with the solvent control were defined as the DMSO group, and HT22 cells treated with MSAB were defined as the MSAB group.

Previous findings have indicated that hypoxic preconditioning (HPC) downregulates GSK-3β expression and activates the GSK-3β/β-catenin signaling pathway, thereby promoting mitochondrial homeostasis and contributing to neuroprotection. However, whether β-catenin, a key downstream effector of GSK-3β, participates in the regulation of mitochondrial homeostasis remains unclear. To address this question, we first pharmacologically inhibited β-catenin, followed by HPC treatment. First, we performed inhibitor concentration screening by CCK8 and detected the reduction of LC3II level after inhibition of β-catenin by LC3II protein (*p* < 0.05; *n* = 3; Fig. S3A–C). The results revealed that cell viability was significantly lower in the β-catenin inhibition + HPC group than in the DMSO and HPC groups (*p* < 0.05; *n* = 3; Fig. 9A). Western blot analysis revealed that under HPC conditions, β-catenin inhibition led to increased DRP1, p-PINK1 and NIX levels, whereas the expression of p-DRP1, MFN2, TOM20, TIM23, LC3, and P62 decreased (*p* < 0.05; *n* = 6; Fig. 9B, C).

We subsequently overexpressed β-catenin via plasmid transfection and subjected the cells to hypoxic conditions. Compared with the vector group, β-catenin overexpression in the hypoxia group enhanced cell viability (*p* < 0.05; *n* = 3; Fig. 9D). Western blot results demonstrated that under HPC conditions, β-catenin overexpression decreased DRP1, p-PINK1 and NIX levels and significantly increased the expression of p-DRP1, MFN2, TOM20, TIM23, LC3, and P62 (*p* < 0.05; *n* = 6; Fig. 9E, F). Immunofluorescence results showed that the TOM20 fluorescence in the overexpression of β-catenin group was higher than that in the simple hypoxia group. Co-localization of LC3 with TOM20 after inhibition of B-catenin showed that mitochondria were more fragmented (Fig. S3F–H). These results suggest that β-catenin contributes to HPC-induced neuroprotection by maintaining the balance of mitochondrial dynamics.

## Discussion

Rapid adaptation to hypoxia is an unanswered question explored by many researchers. HPC, as an endogenous cytoprotective mechanism, is an effective intervention for rapid adaptation to hypoxia. In particular, neuronal cells are the least tolerant to hypoxia. HPC reverses the pathological process through a gentle and appropriate degree of stimulation [4] and can induce the release of endogenous protective substances from the organism, which play important roles in protecting energy metabolism and help to attenuate and resist damage due to ischemia and hypoxia [25]. Therefore, the study of the protective mechanism of HPC on neuronal cells and its molecular mechanism has been a popular research topic. This project reports an endogenous potential mechanism for adaptation to acute hypoxic conditions that cause damage to the organism. Mechanistically, HPC may exert neuroprotective effects by activating the GSK-3β/β-catenin signaling pathway, which in turn regulates mitochondrial homeostasis.

Mitochondrial homeostasis is controlled by different interactions between the processes of mitochondrial fusion and division, mitochondrial biogenesis and degradation (including mitochondrial autophagy), and intracellular transport [8]. The delicate balance between mitochondrial fusion and division is critical for cell survival and optimal function, while mitochondrial autophagy, an endogenous adaptive response, affects neuronal fate in the ischemic brain through selective mitochondrial turnover [26–28]. When mitochondrial fusion is inhibited, mitochondrial fission occurs, which allows the recognition of dysfunctional daughter mitochondria and the subsequent removal of nonmitochondrially functioning daughter mitochondria by lysosomal degradation (i.e., mitochondrial autophagy) [29, 30]. Studies have shown that under normal conditions, autophagy occurs to help cells survive and differentiate, whereas excessive autophagy disrupts biological processes and leads to cell death [31, 32]. In vivo and in vitro studies have shown that compared with hypoxia, HPC reduces the expression of DRP1 protein. Immunofluorescence results revealed that HPC attenuated mitochondrial fission compared with hypoxic treatment, suggesting that HPC reduces excessive mitochondrial fragmentation.

Our study further revealed that, compared with the hypoxia group, the HPC group exhibited elevated protein levels of TOM20 and TIM23, although these levels remained lower than those in the control group. P62 protein levels decreased under both hypoxic and HPC conditions. Co-localization of TOM20 with LC3II showed a greater activation of LC3II under hypoxia. Following chloroquine (CQ) treatment, marked accumulation of LC3II was observed in the hypoxia group, indicating that hypoxia alone induces robust autophagic flux. Upon blockade of autophagic degradation by CQ, the HPC group displayed extensive and compact formation of “autophagosome–mitochondria–lysosome” complexes. These results are also partially consistent with previous results showing that hypoxia regulates mitochondria [33]. Therefore, we speculate that HPC exerts neuroprotective effects by attenuating excessive mitochondrial fission and regulating mitophagy under basal conditions (i.e., by reducing the induction of mitochondrial damage), thereby maintaining mitochondrial homeostasis.

The GSK-3β/β-catenin pathway is a highly conserved classical signaling pathway that is activated by nuclear translocation of β-catenin to control cell proliferation and survival [34]. Glycogen synthase kinase 3 (GSK-3) is a key and common component of the TSC1/mTOR and Wnt signaling pathways and is activated in its nonphosphorylated basal state, whereas Wnt ligand stimulation leads to its phosphorylation and inhibition, which in turn leads to activation of the classical Wnt/β-catenin signaling pathway [20]. The hypoxic conditions of the organism lead to the inability of the extracellular Wnt ligand to bind to its corresponding receptor, which cannot lead to normal activation of the Wnt/β-catenin pathway; thus, the level of GSK-3β phosphorylation decreases, and the level of GSK-3β protein increases. Meanwhile, β-catenin is the substrate of GSK-3β, and GSK-3β-phosphorylated β-catenin can be degraded by the proteasome through ubiquitination [17], resulting in the inability of β-catenin to enter the nuclear fraction properly and inhibiting cell proliferation and survival [35, 36]. Activation of dynamic related protein 1 (DRP1) via the Wnt/GSK-3β/β-catenin signaling pathway has also been shown to mediate apoptosis associated with excessive mitochondrial division and endoplasmic reticulum (ER) stress [37]. GSK-3β is involved in a myriad of pathways throughout the CNS, depending on the phosphorylation site, which can be neuroprotective or neurodegenerative, and GSK-3β activity is increased by the phosphorylation of tyrosine-216 and decreased by the phosphorylation of serine-9 [38]. A large body of evidence suggests that GSK-3β inhibits tyrosine-216 dephosphorylation and reduces neuronal apoptosis, and our results confirm that HPC may play a protective role by activating the GSK-3β/β-catenin signaling pathway.

We conducted both in vivo and in vitro experiments and reported that the protein level of GSK-3β was significantly reduced after HPC treatment, while the protein levels of p-GSK-3β and the downstream protein β-catenin were significantly increased; i.e., the GSK-3β protein was phosphorylated after HPC, which reduced the involvement of the β-catenin degradation process, thus activating the GSK-3β/β-catenin signaling pathway, affecting downstream factors and exerting protective effects. Wnt activation can stimulate protein translation through the inactivation of GSK-3 and TSC1/2, which in turn activates mTOR [39]. TSC1 plays important roles in development, cell growth and proliferation, survival, autophagy and cilia development through its cooperation with multiple regulatory molecules [40]. This finding was verified by in vitro experiments in which the addition of GSK-3β inhibitor significantly increased the β-catenin protein level and activated the Wnt/β-catenin signaling pathway. In addition, the TSC1 protein level was also significantly increased. However, the downstream regulatory mechanism of the Wnt/GSK-3β/β-catenin pathway remains unclear and warrants further investigation.

HPC may alleviate hypoxia-induced neural injury by inducing a mild degree of mitochondrial damage, activating the GSK-3β/β-catenin signaling pathway, and modulating mitochondrial fission and fusion while maintaining mitochondrial homeostasis, accompanied by corresponding changes in mitophagy markers. To further validate whether HPC regulates mitochondrial homeostasis through activation of the GSK-3β/β-catenin signaling pathway, we performed reverse validation experiments using a GSK-3β inhibitor and transfection with a GSK-3β overexpression plasmid in vitro. The results showed that following GSK-3β inhibition, the GSK-3β/β-catenin signaling pathway was activated, mitochondrial fragmentation was markedly attenuated, and mitophagy-related markers (e.g., p-PINK1, NIX) were correspondingly reduced. Treatment of hypoxia-stimulated cells with the GSK-3β inhibitor reversed the hypoxia-induced changes in mitophagy markers. Upon GSK-3β overexpression, HPC-treated cells exhibited increased mitochondrial fission compared with the empty vector group, accompanied by enhanced changes in mitophagy markers. In contrast, reverse validation experiments using a β-catenin inhibitor and β-catenin overexpression plasmid yielded opposite effects to those observed with GSK-3β manipulation.

It should be noted that the above data establish a central role for the GSK-3β/β-catenin signaling pathway in regulating mitochondrial fission and homeostasis—a primary outcome validated in this study. However, the relationship between β-catenin and mitophagy currently remains correlative rather than causal. Our data show that changes in β-catenin signaling are consistently associated with alterations in mitophagy markers; nevertheless, it is worth noting that existing literature has revealed a potential link between β-catenin and mitophagy. For instance, studies have shown that autophagy can promote β-catenin nuclear translocation by activating HDAC6, thereby regulating mitochondrial energy metabolism [41]. Additionally, following its release from damaged mitochondria, Pgam5 can activate Wnt/β-catenin signaling by interacting with Axin, thereby inducing mitochondrial biogenesis [42]. These findings suggest the existence of a regulatory network between β-catenin and mitophagy that has yet to be fully elucidated.

However, this study does not provide mechanistic evidence for direct regulation of mitophagy by β-catenin (e.g., transcriptional regulation of mitophagy-related genes or direct interaction with autophagic proteins). Moreover, the chloroquine flux assay employed in this study reflects total autophagic flux rather than mitophagy-specific flux. Therefore, the results suggest that HPC modulates mitochondrial fission and homeostasis via the GSK-3β/β-catenin pathway, with alterations in the activity of this pathway being associated with changes in mitophagy markers. Mitophagy serves as a potential accompanying process, and its direct regulatory relationship, as well as mitochondria-specific flux, require further validation in future studies.

Taken together, these findings suggest that HPC may exert neuroprotective effects by activating the GSK-3β/β-catenin signaling pathway and downstream protective factors to maintain mitochondrial homeostasis. These findings confirm that the GSK-3β/β-catenin signaling pathway regulates mitochondrial homeostasis (e.g., mitochondrial fission and fusion) and is a potential target for HPC neuroprotective therapy.

## Conclusions

Our integrated in vivo and in vitro experiments demonstrated that hypoxic preconditioning (HPC) protects against hypoxic injury in the mouse hippocampus by maintaining mitochondrial homeostasis via the GSK-3β/β-catenin signaling pathway. Mechanistically, HPC alleviates pathological mitochondrial fission by inhibiting GSK-3β and increasing β-catenin activity, while also promoting adaptive mitophagy and reducing apoptosis. Under hypoxic conditions, pharmacological inhibition of GSK-3β or overexpression of β-catenin recapitulated the effects of HPC, significantly reducing mitochondrial fission. Conversely, overexpression of GSK-3β and pharmacological inhibition of β-catenin reversed the HPC-mediated inhibition of mitochondrial fission, confirming the regulatory role of this pathway (Fig. 9G). These findings establish the critical role of the GSK-3β/β-catenin signaling pathway in the mitochondrial quality control mechanism of HPC, providing new mechanistic insights into hypoxia-induced neuroprotection.

## Supplementary information

Supplementary figure legends

Fig. S1 Schematic diagram of the technical route process.

Fig. S2 Effects of GSK-3β overexpression and inhibition of GSK-3β on mitochondrial homeostasis.

Fig. S3 Effects of β-catenin overexpression and β-catenin inhibition on mitochondrial homeostasis.

Fig. S4 Effects of HPC on mitophagic flux.

original date

Cell Insight CX7 HCS platform assay

## Data Availability

The data that support the findings of this study are available from the corresponding author upon reasonable request. Western blots will be published if your article is accepted for publication.
