# Supplementary material for: HPC restores mitochondrial homeostasis and ameliorates hypoxic stress through the GSK-3β/β-catenin signaling pathway
Source: Cell Death Dis. 2026 Jul 8;17(1):853. doi: 10.1038/s41419-026-09080-7 (PMC13631344; doi:10.1038/s41419-026-09080-7)
Supplement: Supplementary file 1 [file 41419_2026_9080_MOESM6_ESM.pdf]

Figure 2

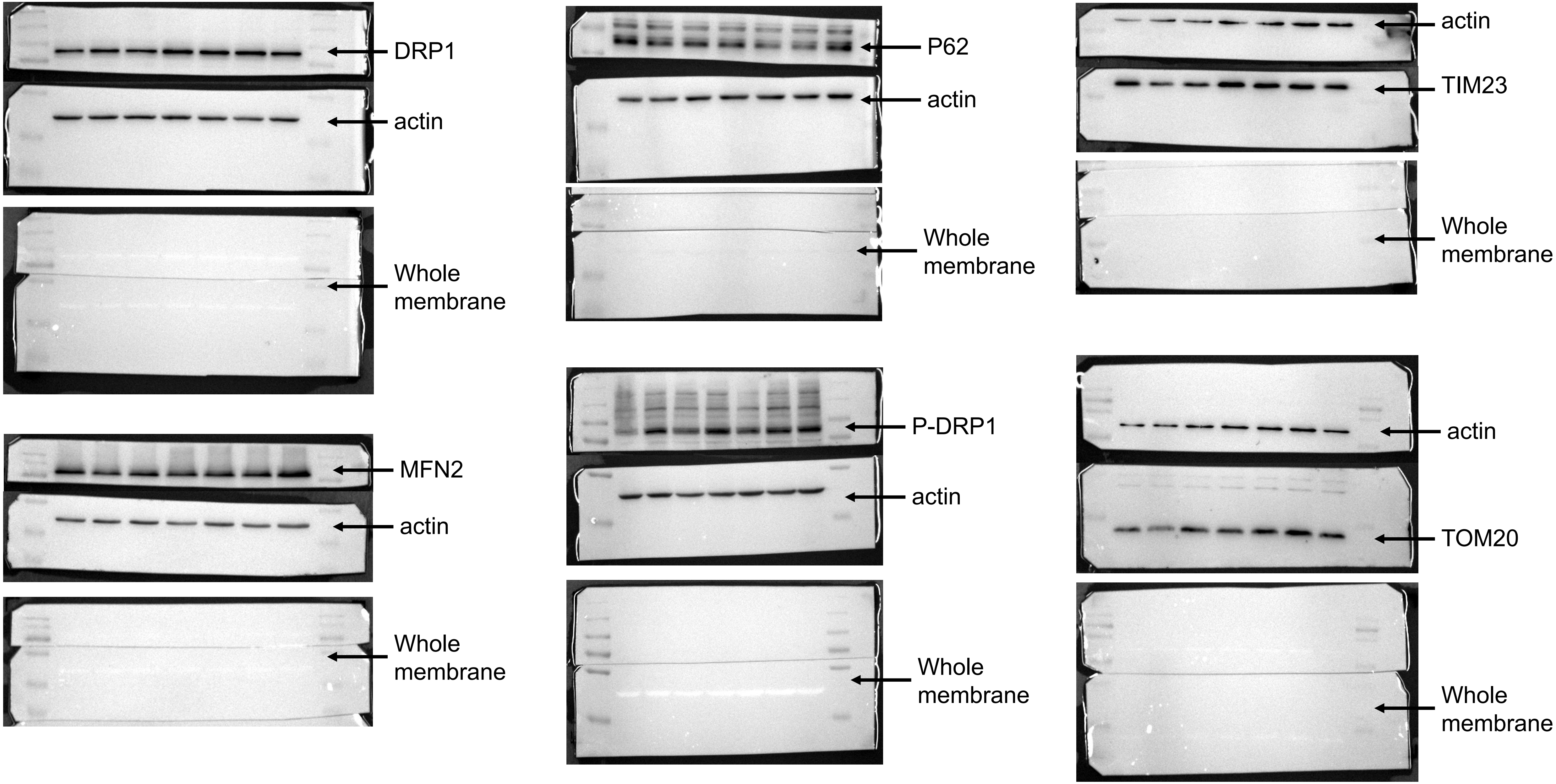

DRP1
actin
Whole membrane
MFN2
actin
Whole membrane
P62
actin
Whole membrane
P-DRP1
actin
Whole membrane
actin
TIM23
Whole membrane
actin
TOM20
Whole membrane

Figure 4

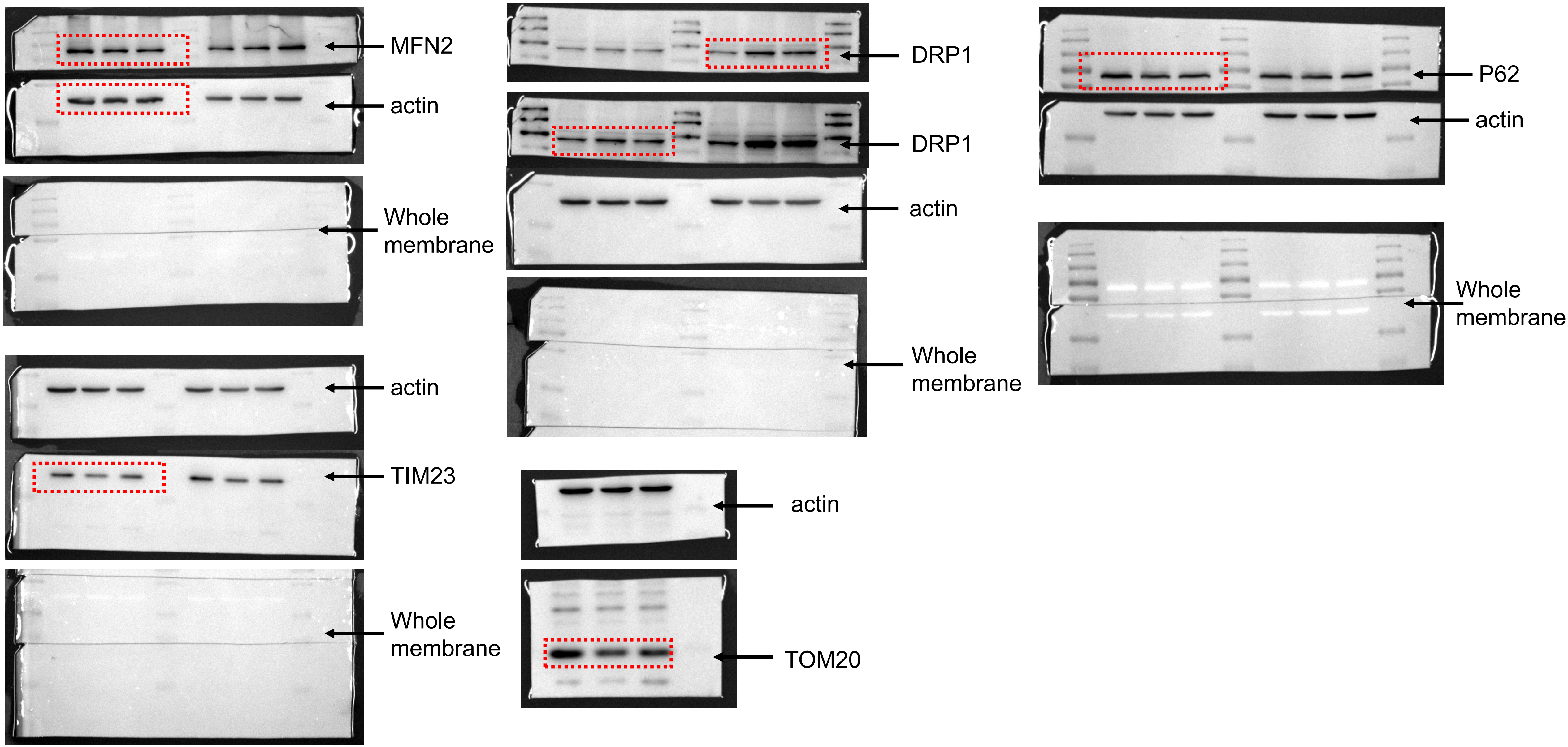
MFN2
actin
Whole membrane
actin
TIM23
Whole membrane
DRP1
DRP1
actin
Whole membrane
actin
TOM20
P62
actin
Whole membrane

Figure 5

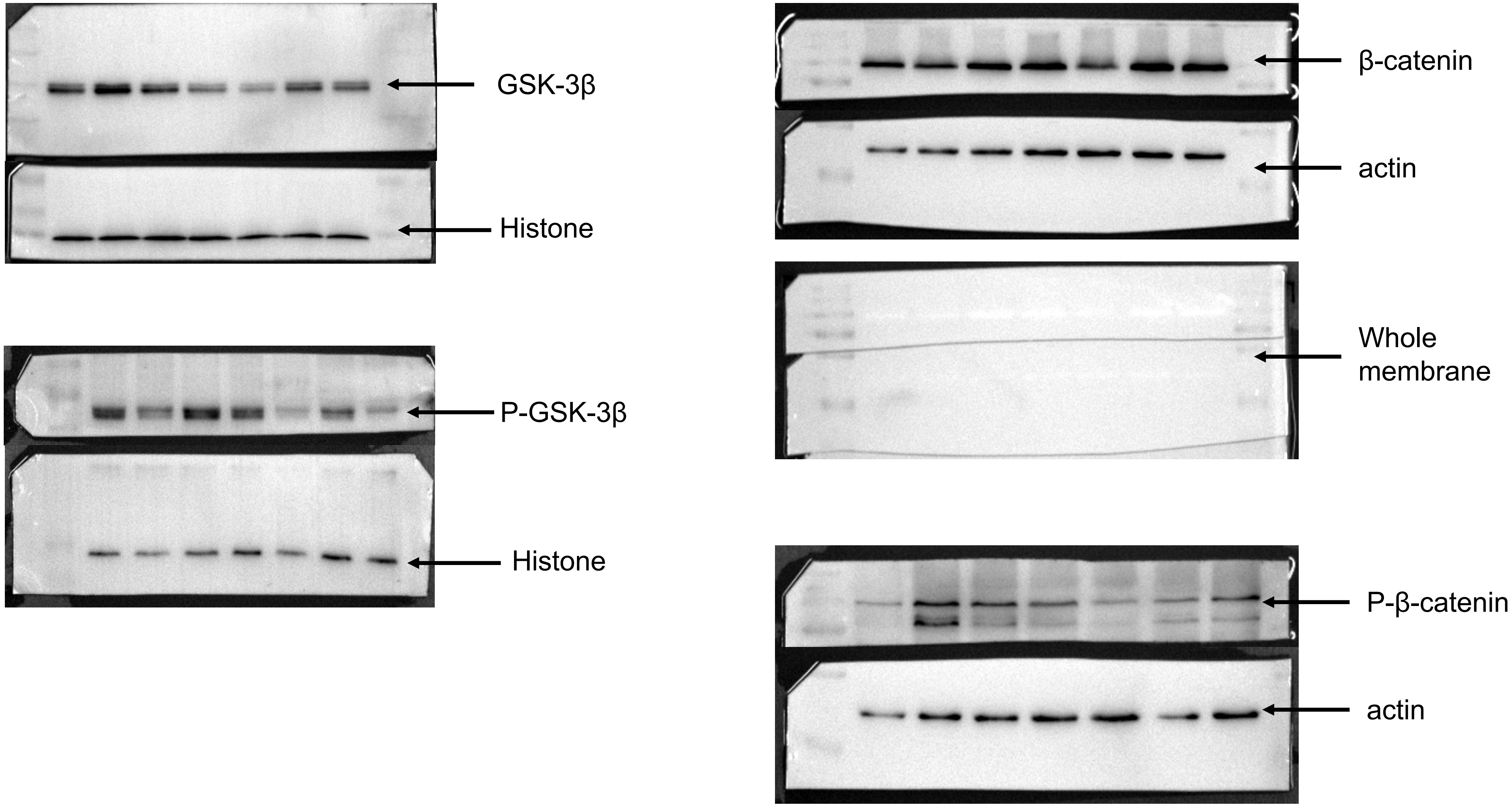
GSK-3β
Histone
P-GSK-3β
Histone
β-catenin
actin
Whole
membrane
P-β-catenin
actin

Figure 6

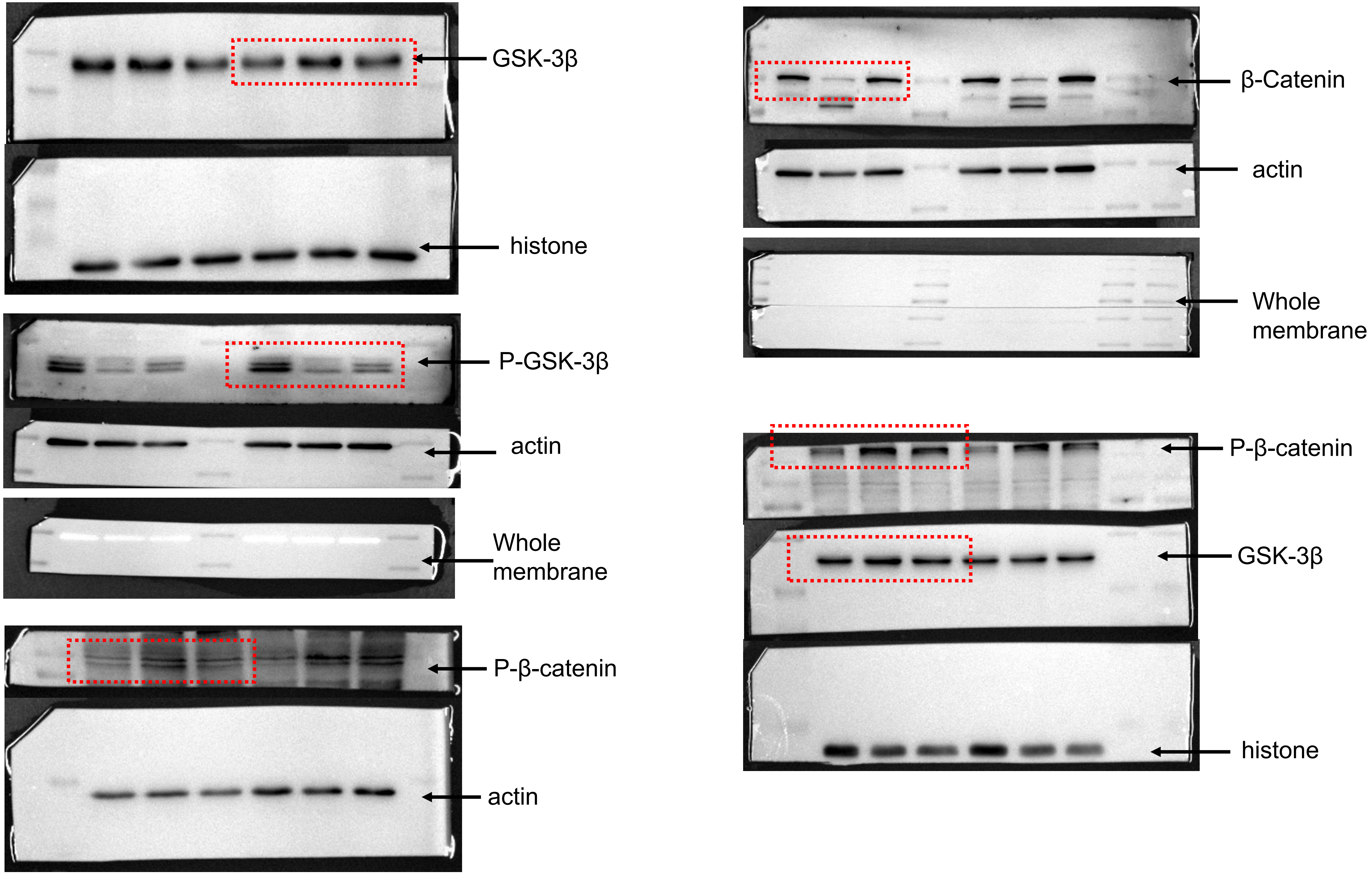
GSK-3β
histone
P-GSK-3β
actin
Whole
membrane
P-β-catenin
actin
β-Catenin
actin
Whole
membrane
P-β-catenin
GSK-3β
histone

Figure 7

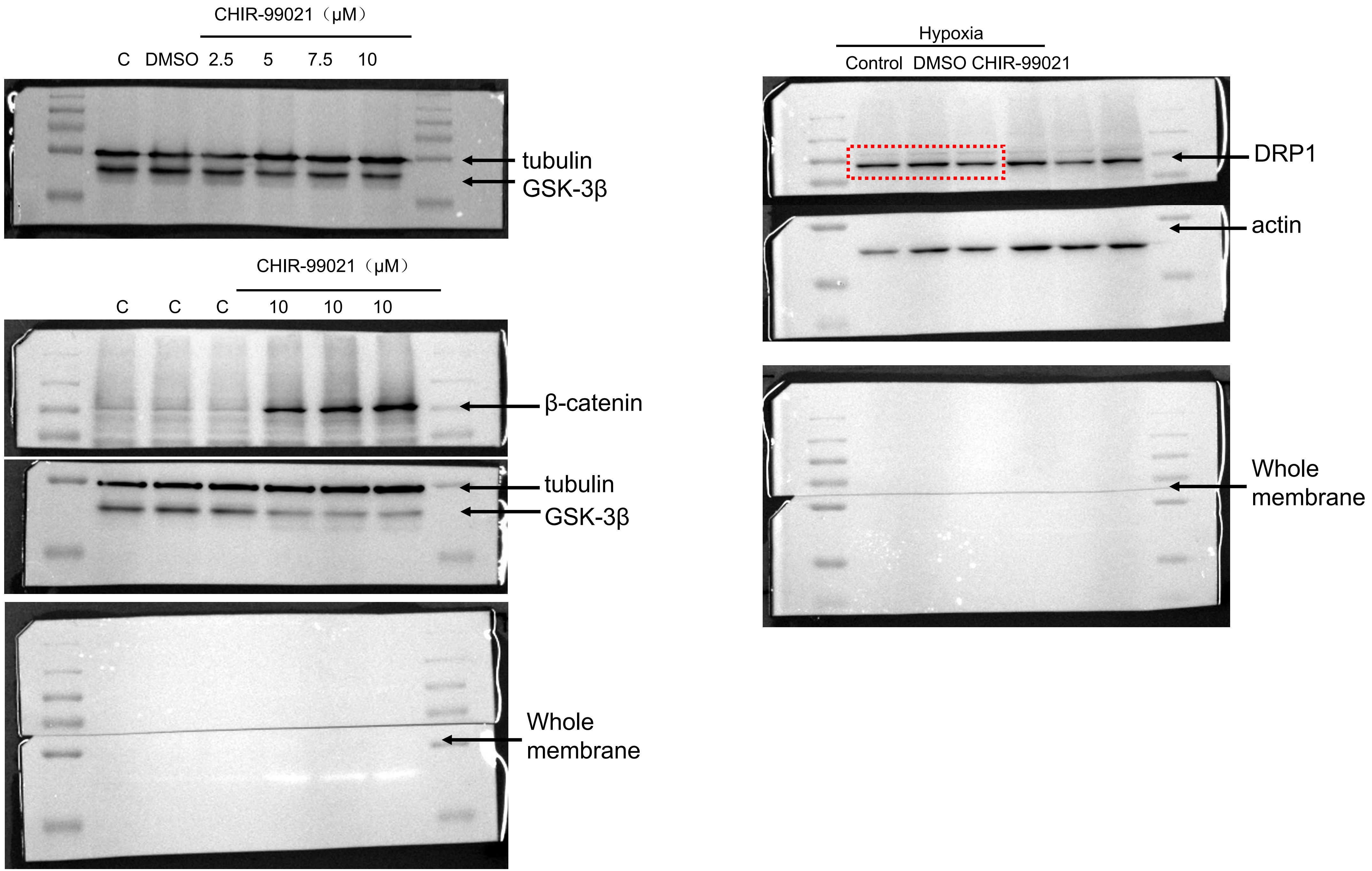

CHIR-99021（μM）
C
DMSO
2.5
5
7.5
10
tubulin
GSK-3β
CHIR-99021（μM）
C
C
C
10
10
10
β-catenin
tubulin
GSK-3β
Whole membrane
Hypoxia
Control
DMSO
CHIR-99021
DRP1
actin
Whole membrane

Figure 7

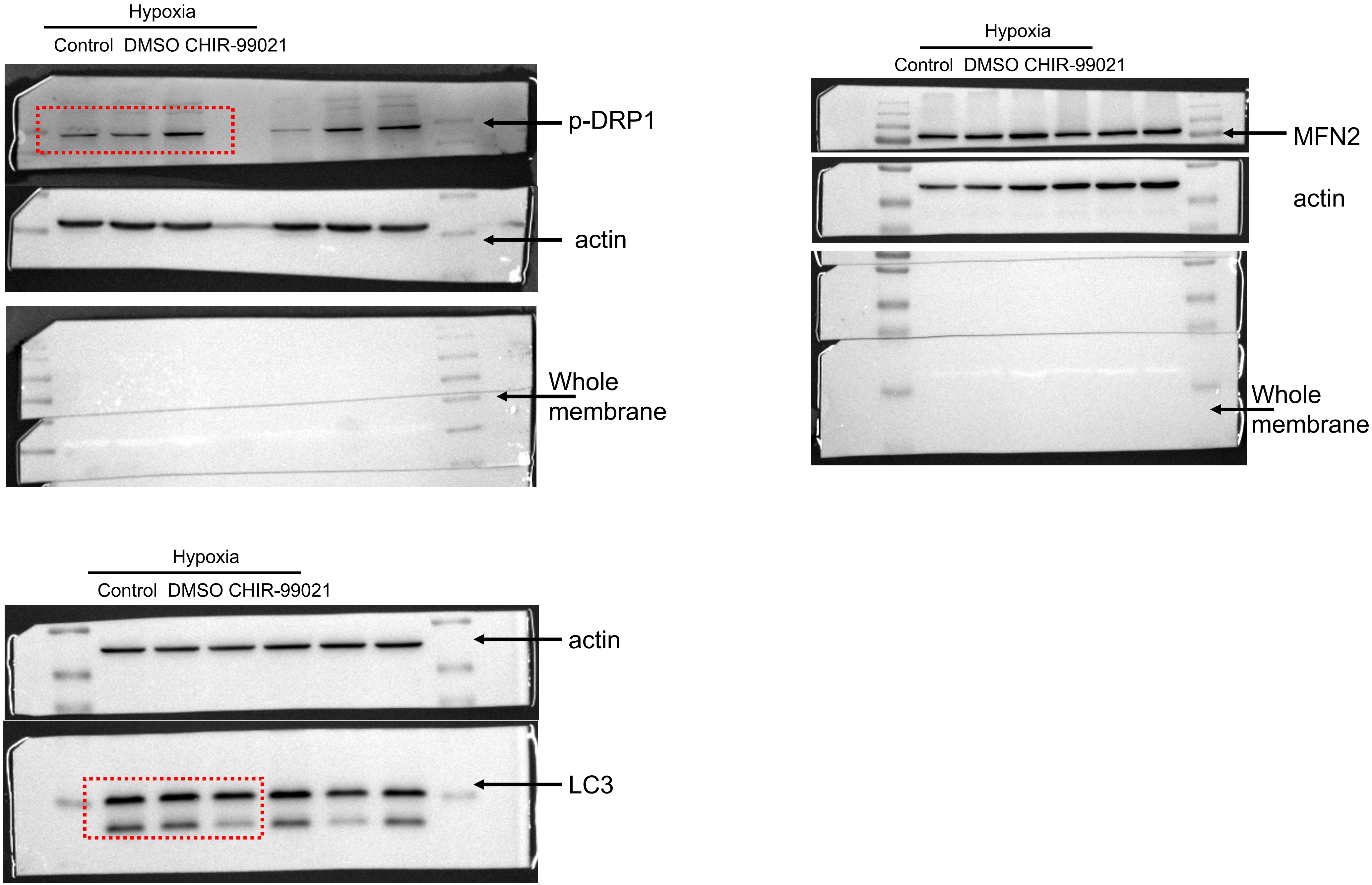

Hypoxia
Control DMSO CHIR-99021
p-DRP1
actin
Whole membrane
Hypoxia
Control DMSO CHIR-99021
MFN2
actin
Whole membrane
Hypoxia
Control DMSO CHIR-99021
actin
LC3

Figure 7

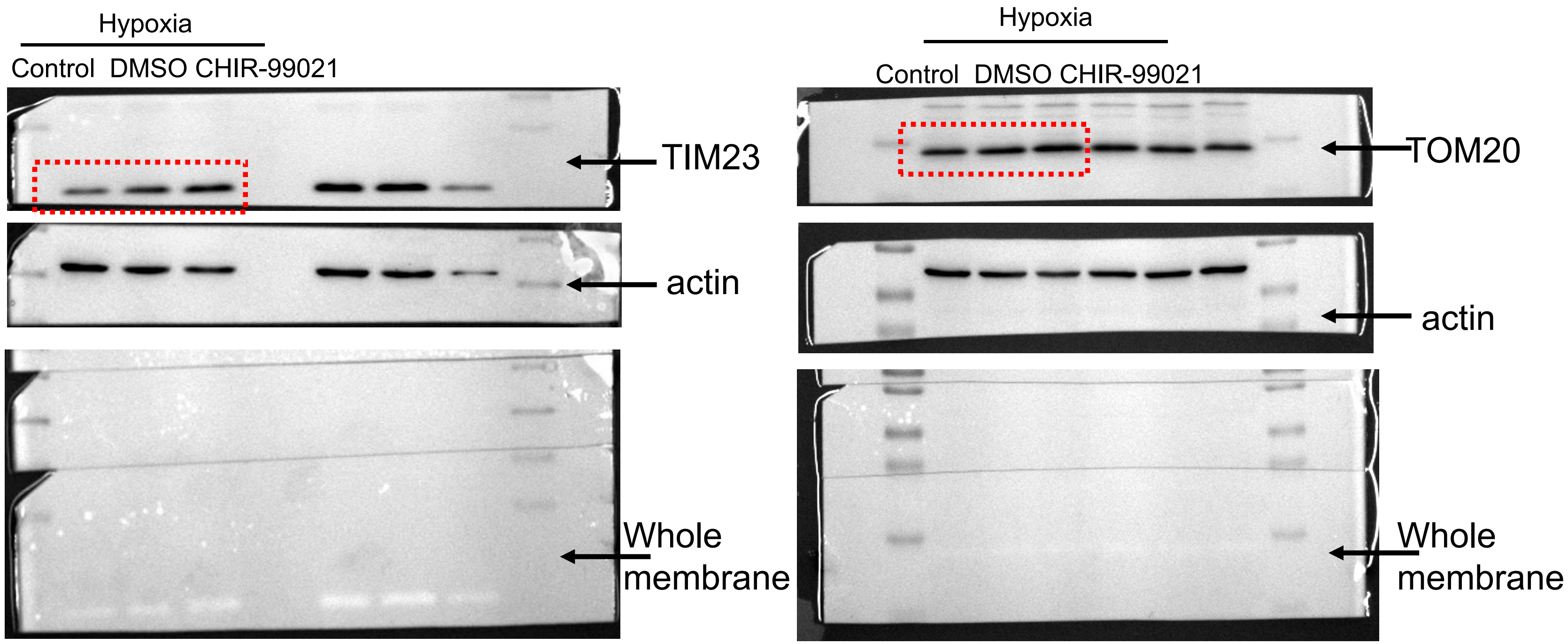

Hypoxia
Control DMSO CHIR-99021
TIM23
actin
Whole membrane
Hypoxia
Control DMSO CHIR-99021
TOM20
actin
Whole membrane

Figure 8

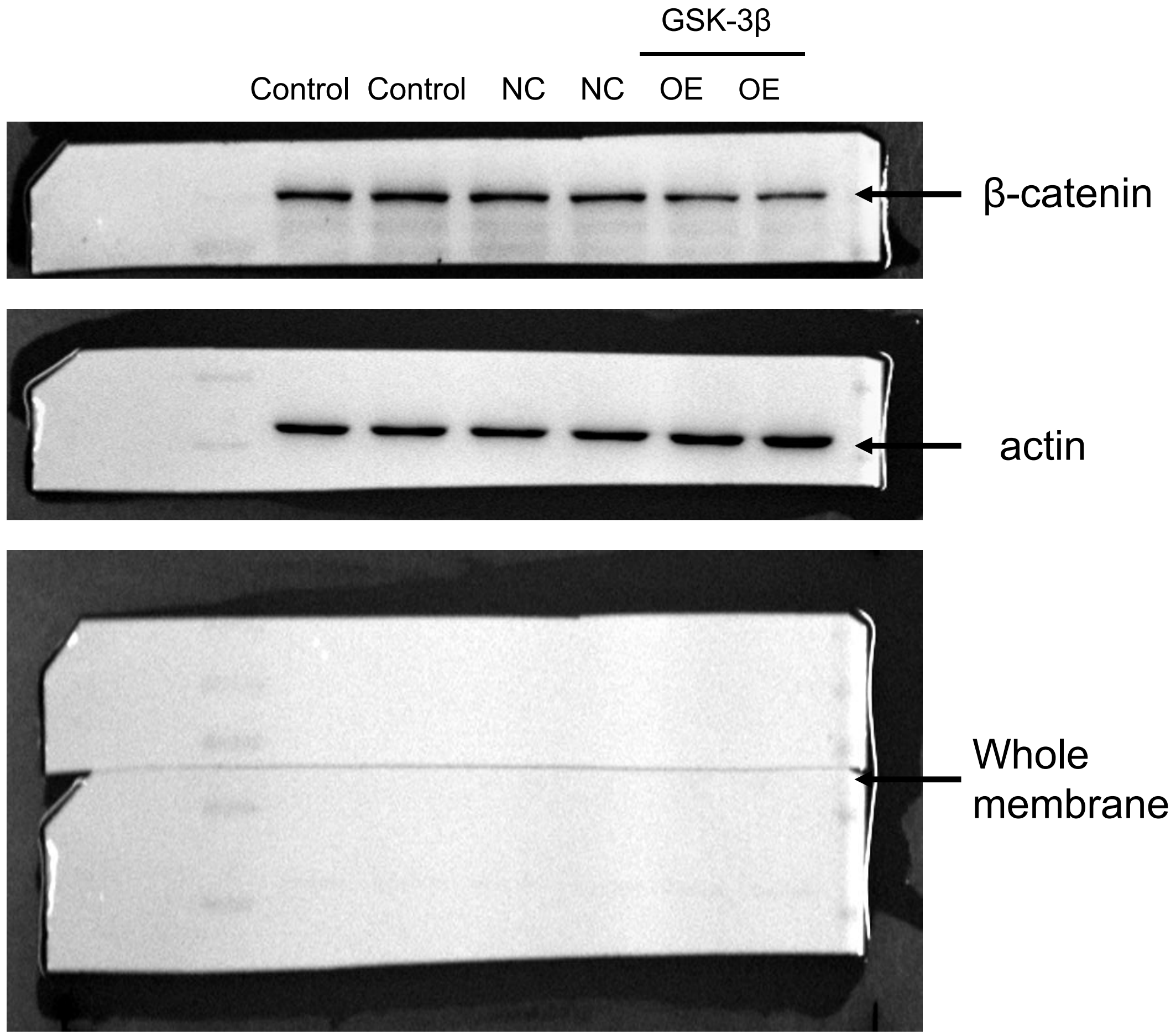
GSK-3β
Control Control NC NC OE OE
β-catenin
actin
Whole membrane

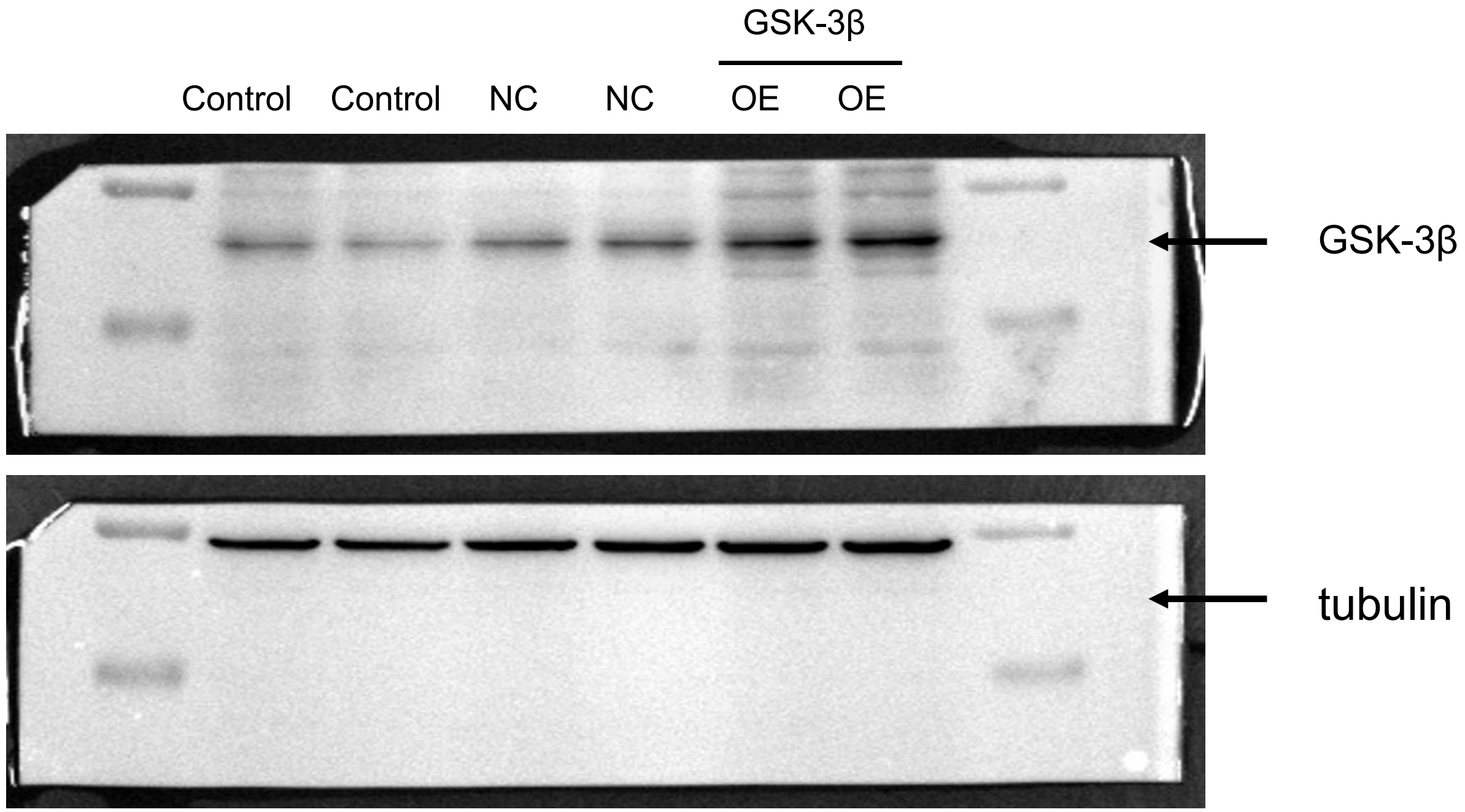
GSK-3β
Control Control NC NC OE OE
GSK-3β
tubulin

Figure 8

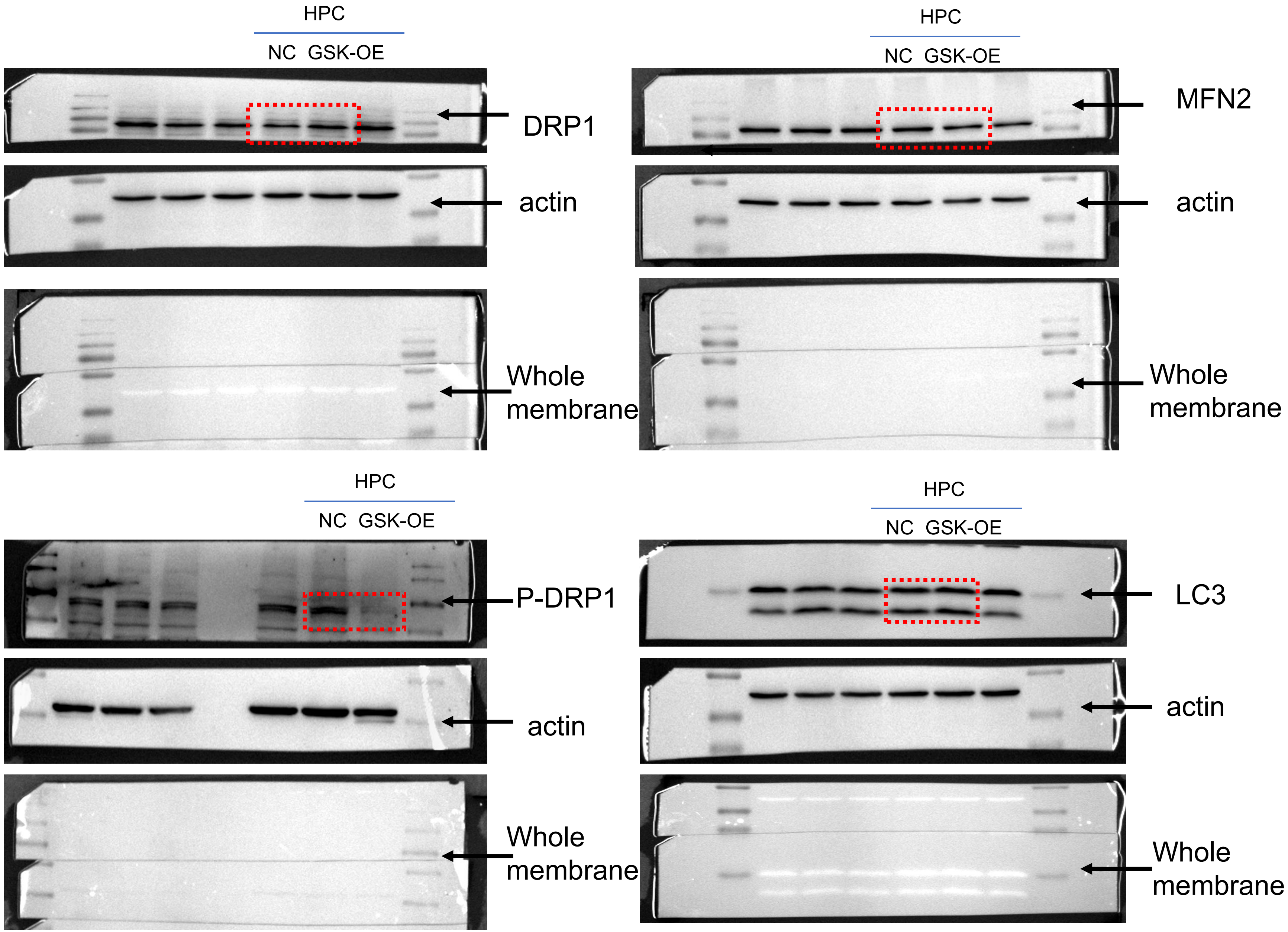

HPC
NC GSK-OE
DRP1
actin
Whole membrane
HPC
NC GSK-OE
MFN2
actin
Whole membrane
HPC
NC GSK-OE
P-DRP1
actin
Whole membrane
HPC
NC GSK-OE
LC3
actin
Whole membrane

Figure 8

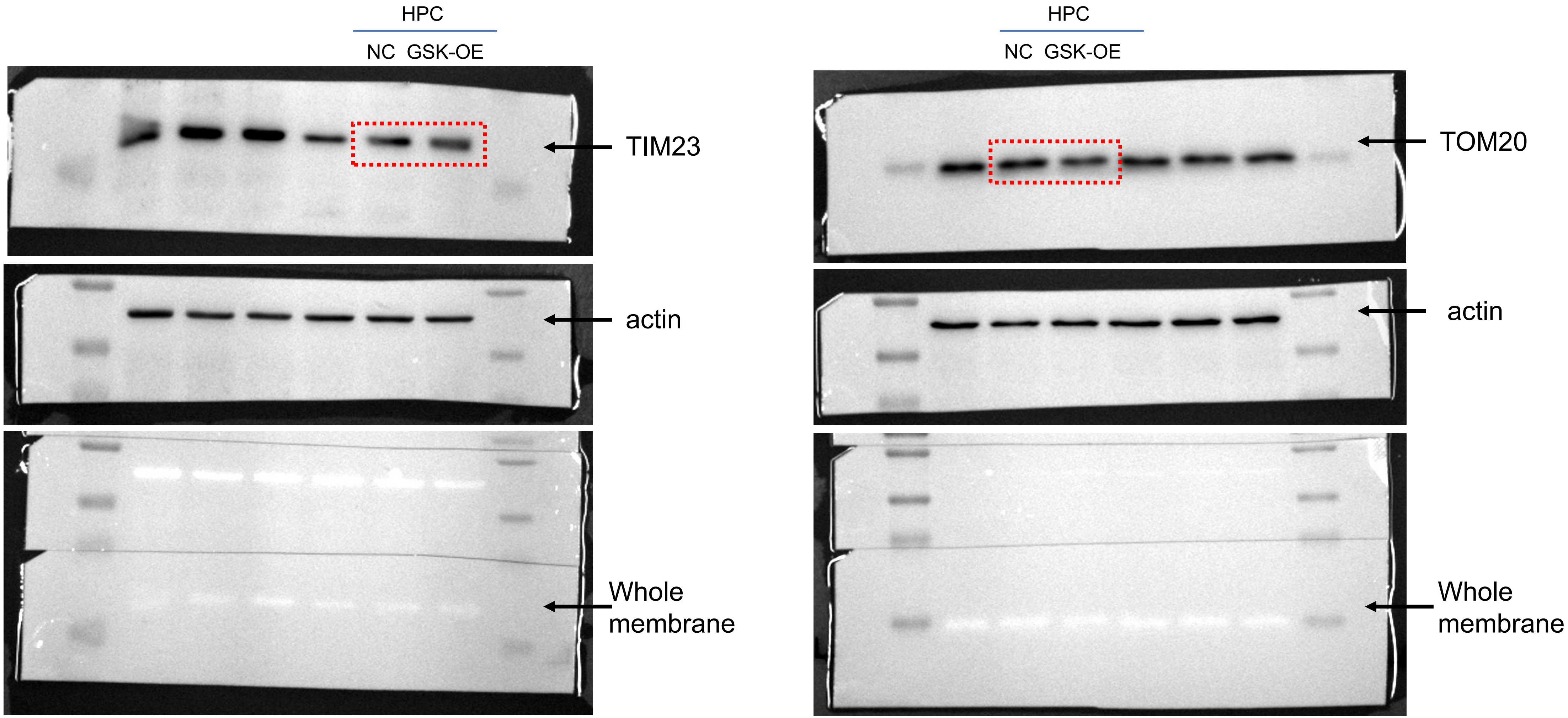
HPC
NC GSK-OE
TIM23
actin
Whole membrane
HPC
NC GSK-OE
TOM20
actin
Whole membrane

Figure 9

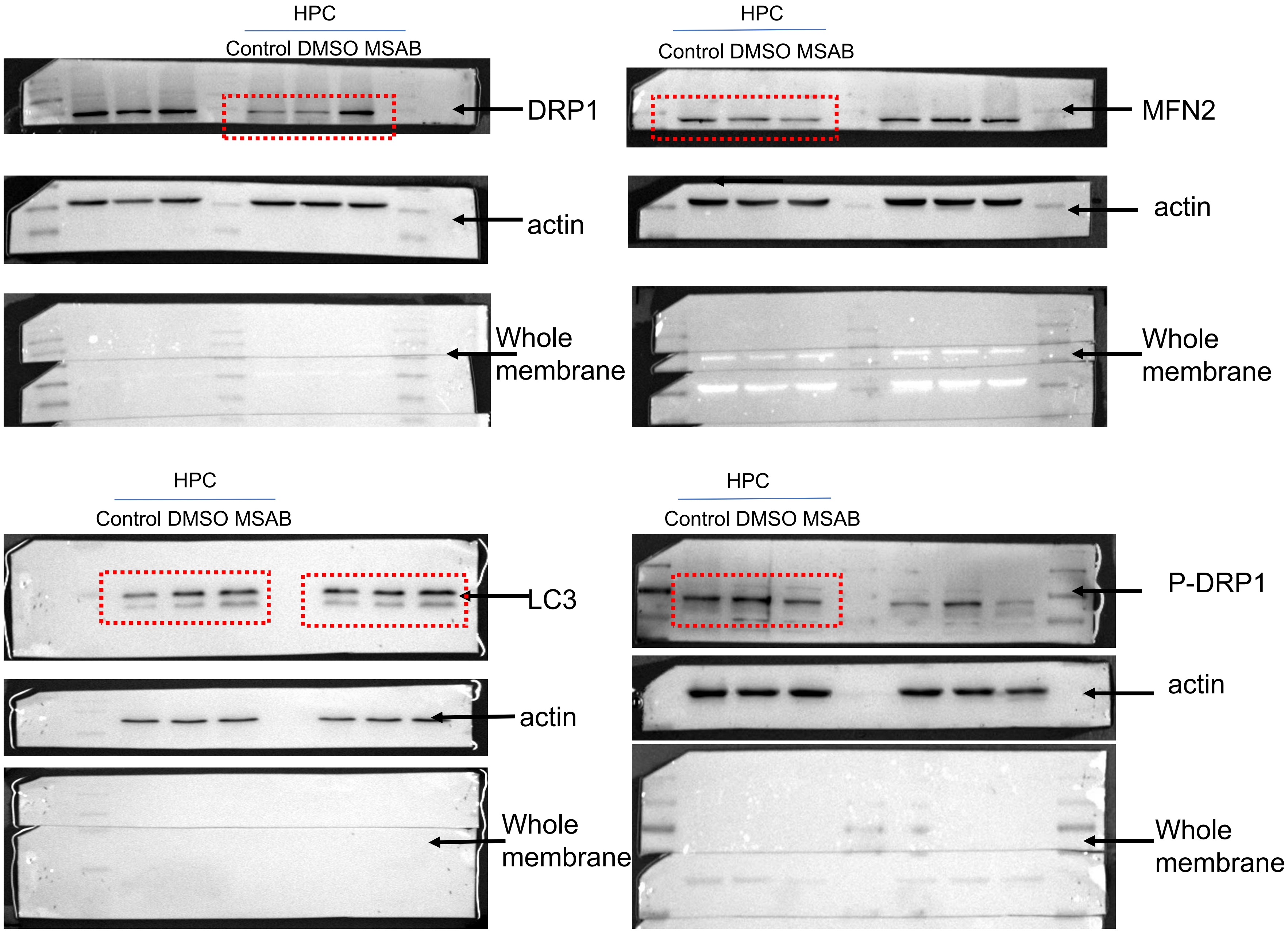
HPC
Control DMSO MSAB
DRP1
actin
Whole membrane
HPC
Control DMSO MSAB
MFN2
actin
Whole membrane
HPC
Control DMSO MSAB
LC3
actin
Whole membrane
HPC
Control DMSO MSAB
P-DRP1
actin
Whole membrane

Figure 9

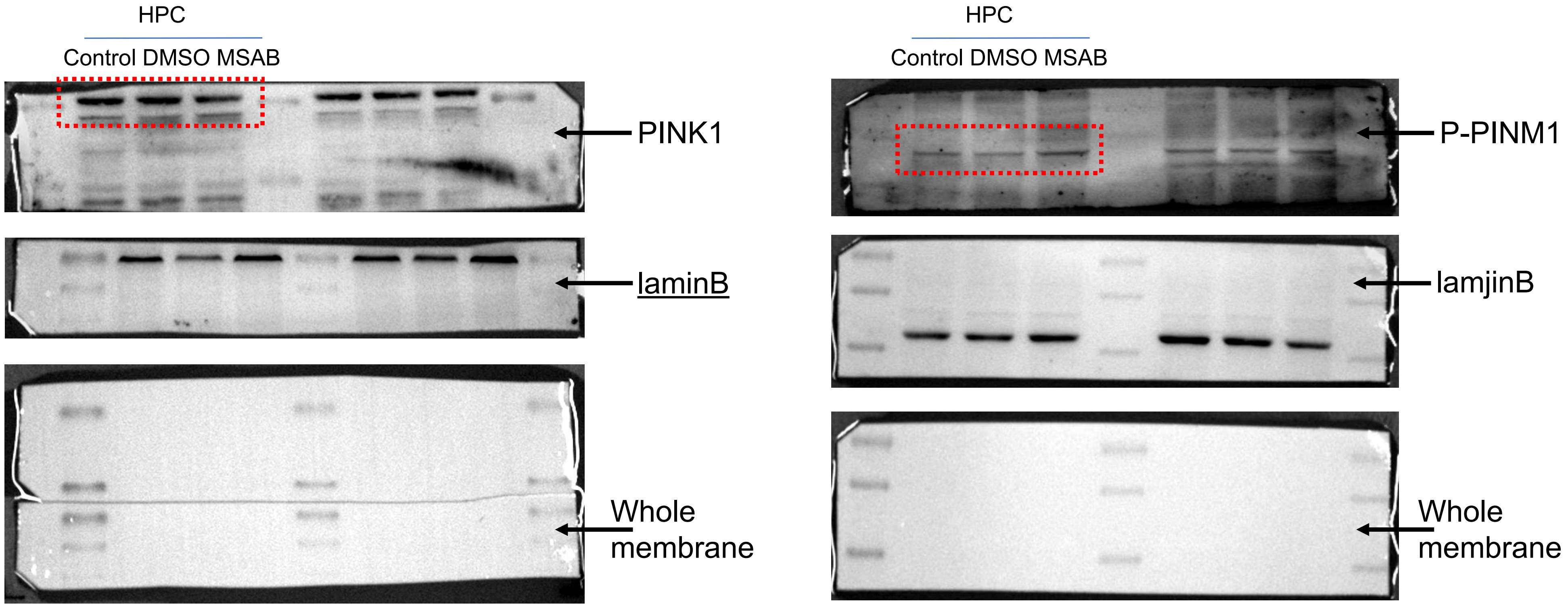
HPC
Control DMSO MSAB
PINK1
laminB
Whole
membrane
HPC
Control DMSO MSAB
P-PINM1
lamjinB
Whole
membrane

Figure 9

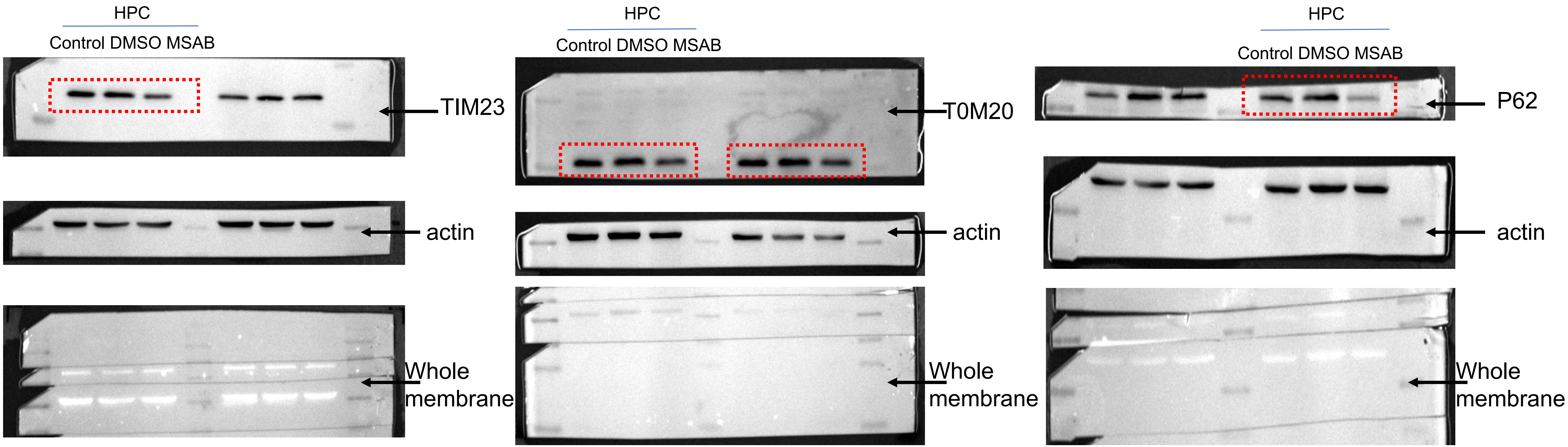
HPC
Control DMSO MSAB
TIM23
actin
Whole membrane
HPC
Control DMSO MSAB
T0M20
actin
Whole membrane
HPC
Control DMSO MSAB
P62
actin
Whole membrane

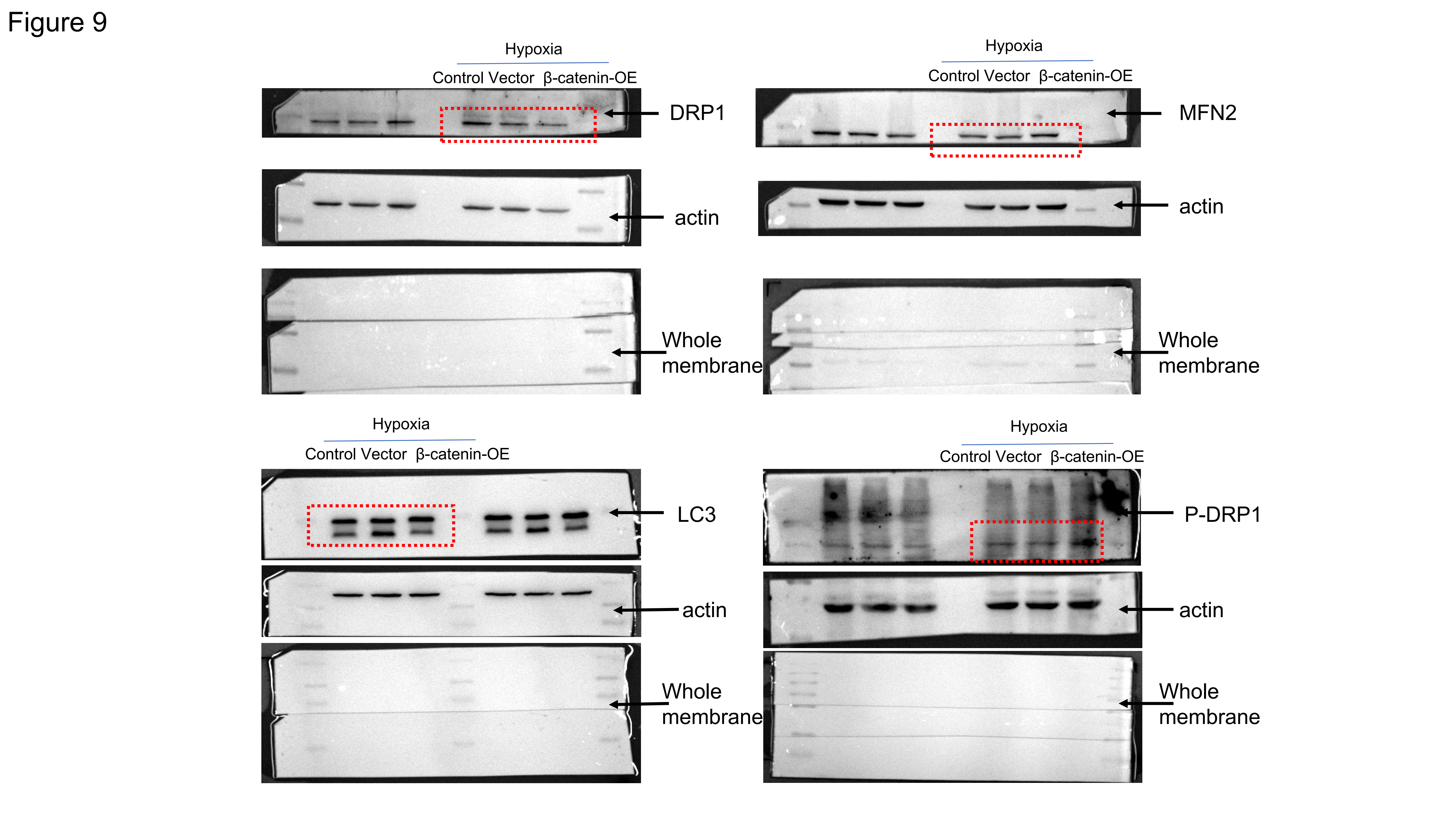
Figure 9
Hypoxia
Control Vector β-catenin-OE
DRP1
actin
Whole membrane
Hypoxia
Control Vector β-catenin-OE
MFN2
actin
Whole membrane
Hypoxia
Control Vector β-catenin-OE
LC3
actin
Whole membrane
Hypoxia
Control Vector β-catenin-OE
P-DRP1
actin
Whole membrane

Figure 9

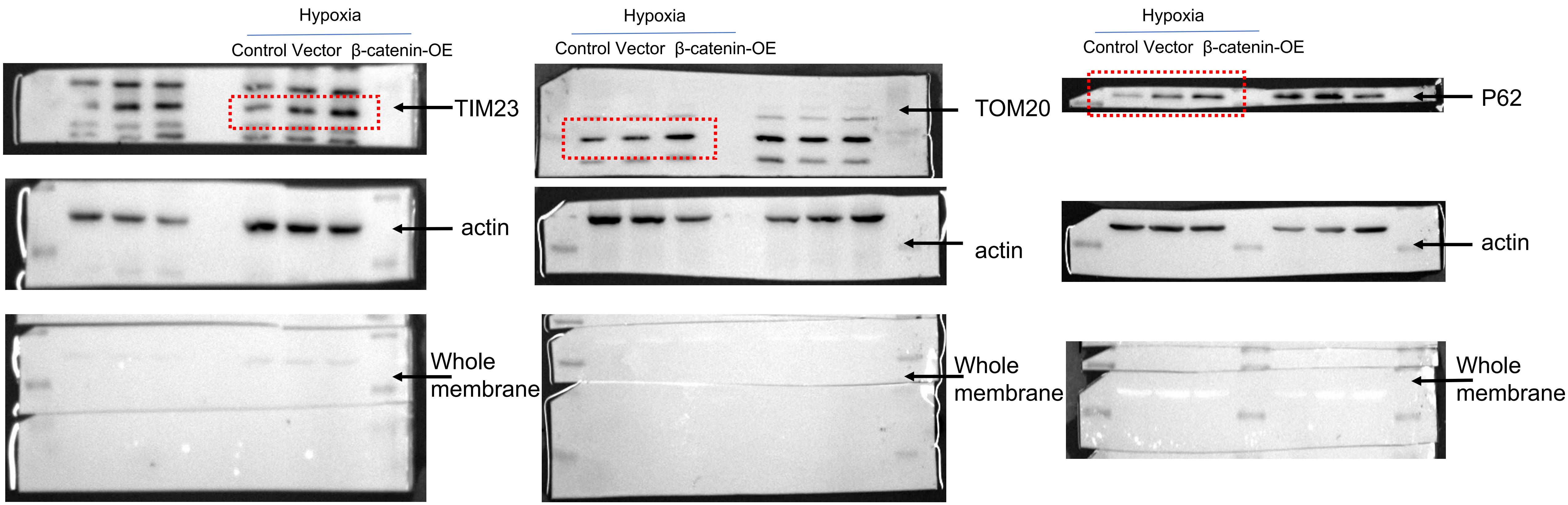
Hypoxia
Control Vector β-catenin-OE
TIM23
actin
Whole membrane
Hypoxia
Control Vector β-catenin-OE
TOM20
actin
Whole membrane
Hypoxia
Control Vector β-catenin-OE
P62
actin
Whole membrane

Figure 9

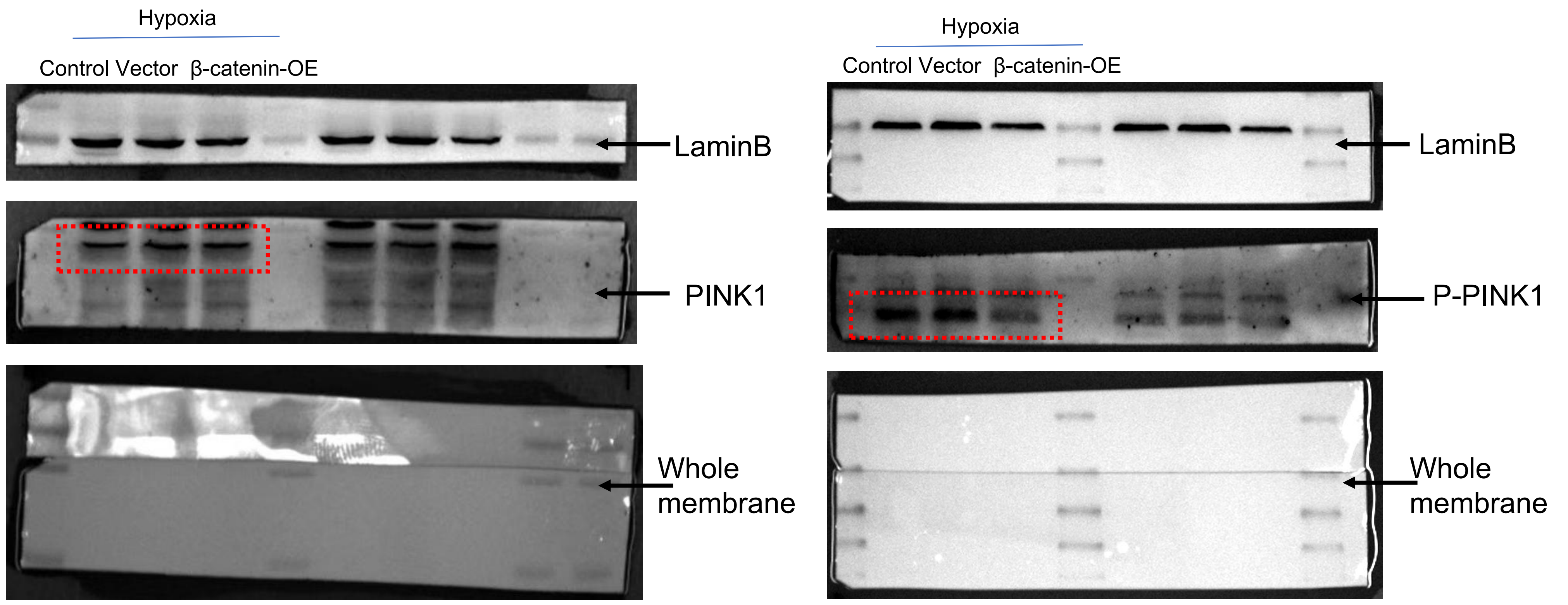
Hypoxia
Control Vector β-catenin-OE
LaminB
PINK1
Whole membrane
Hypoxia
Control Vector β-catenin-OE
LaminB
P-PINK1
Whole membrane

Figure 9

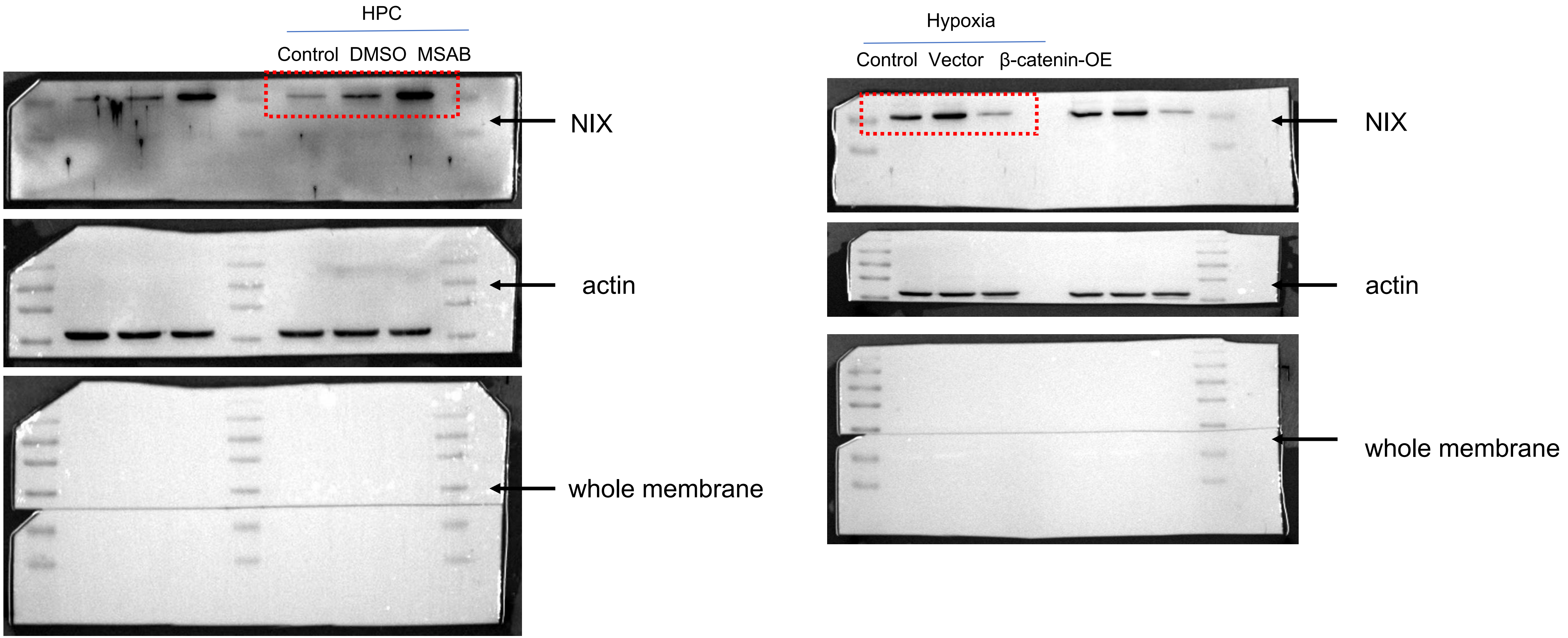
HPC
Control
DMSO
MSAB
NIX
actin
whole membrane
Hypoxia
Control
Vector
β-catenin-OE
NIX
actin
whole membrane

Figure S2

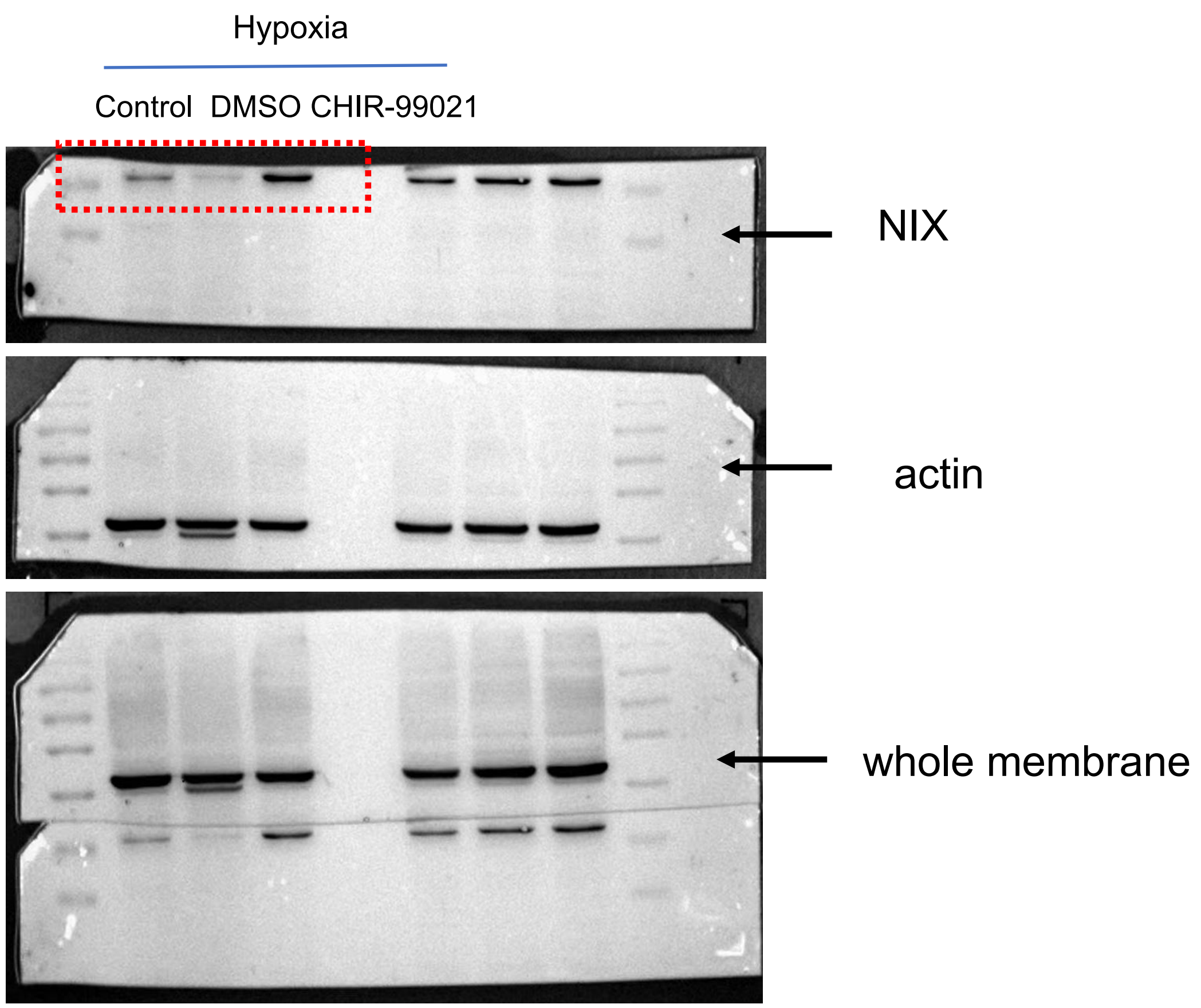
Hypoxia
Control DMSO CHIR-99021
NIX
actin
whole membrane

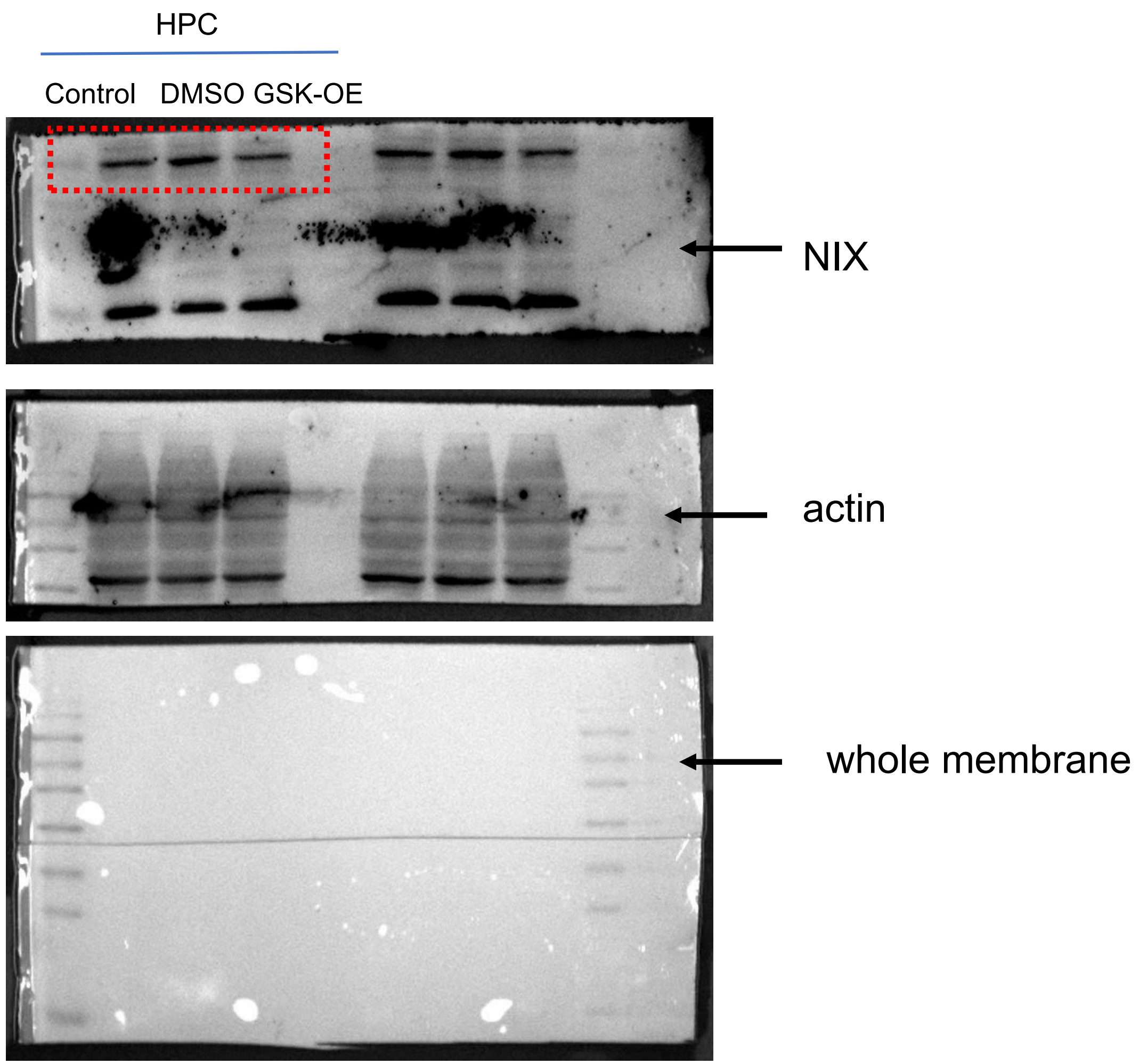
HPC
Control DMSO GSK-OE
NIX
actin
whole membrane

Figure S3

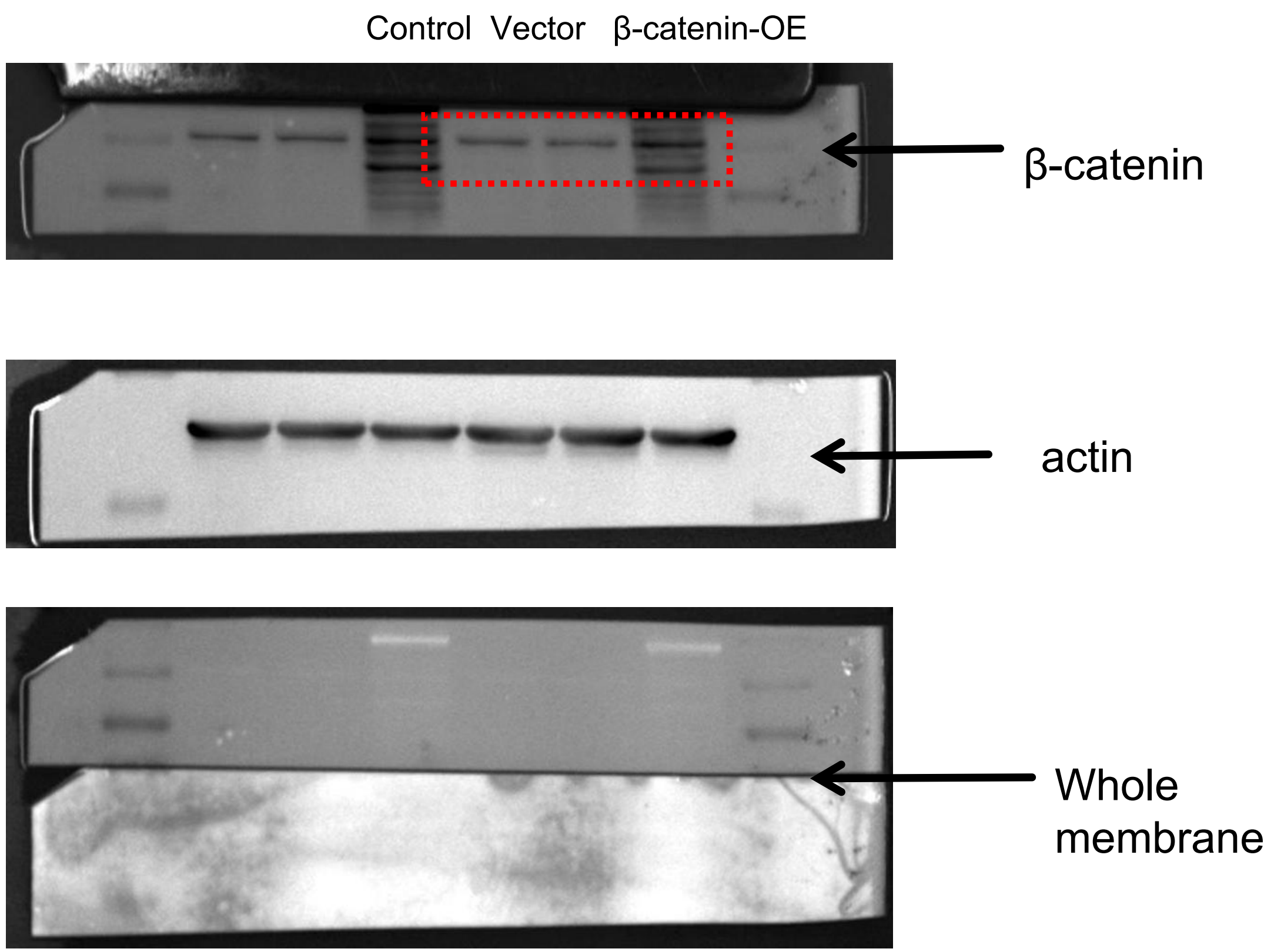
Control Vector β-catenin-OE
β-catenin
actin
Whole membrane

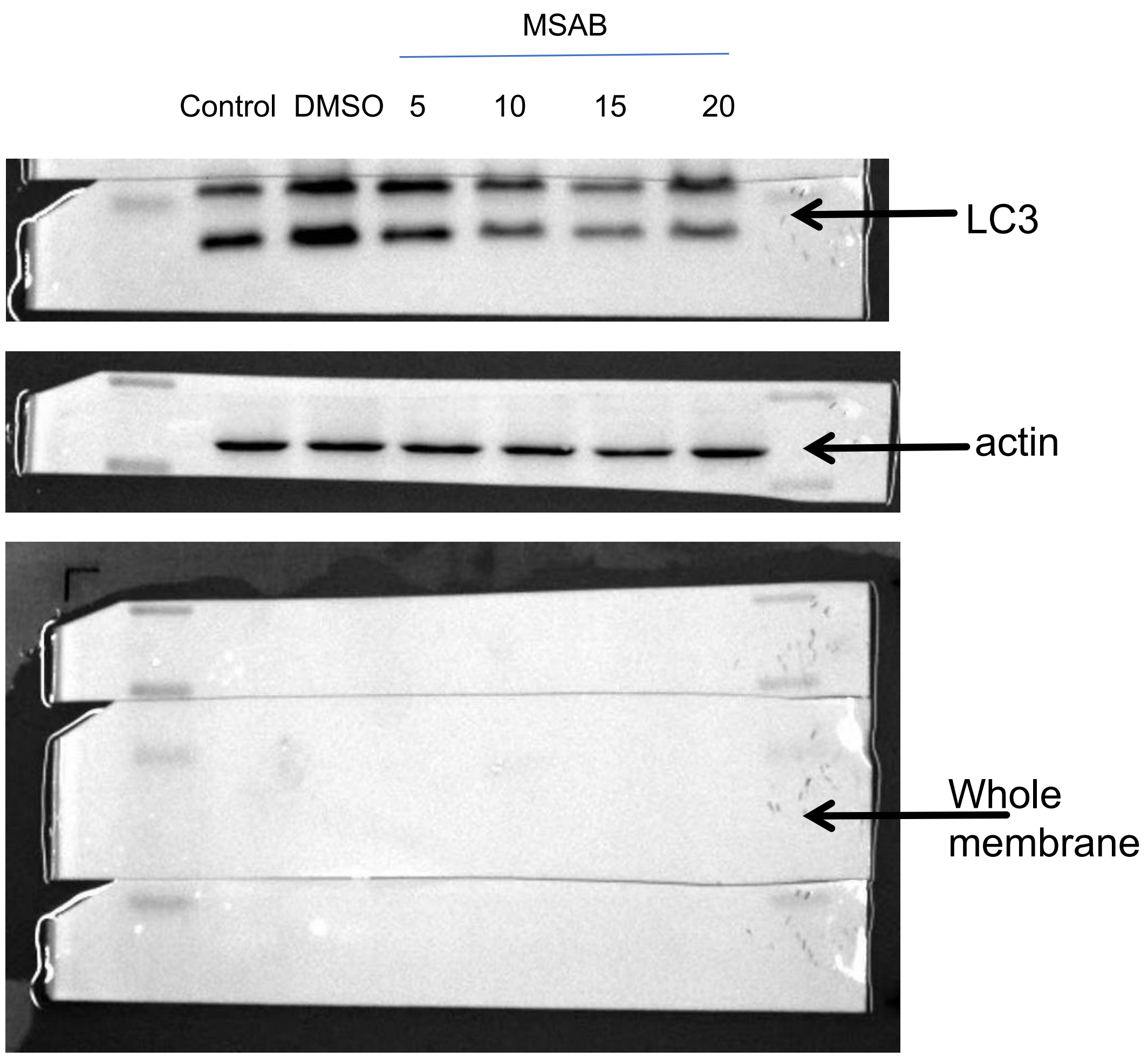
MSAB
Control DMSO 5 10 15 20
LC3
actin
Whole membrane
