# Supplementary material for: HPC restores mitochondrial homeostasis and ameliorates hypoxic stress through the GSK-3β/β-catenin signaling pathway
Source: Cell Death Dis. 2026 Jul 8;17(1):853. doi: 10.1038/s41419-026-09080-7 (PMC13631344; doi:10.1038/s41419-026-09080-7)
Supplement: Supplementary file 2 [file 41419_2026_9080_MOESM1_ESM.docx]

**Fig. S1** **Schematic diagram of the technical route process.**

**Fig. S2** **Effects of GSK-3β overexpression and inhibition of GSK-3β on mitochondrial homeostasis.**

A. Fluorescence and bright-field imaging of GSK-3β-OE and Vector transfected cells; B. Representative protein band of mitophagy-related proteins; C. Quantification of PINK1, p-PINK1 and NIX (n=6); D. Representative protein band of mitophagy-related proteins; E. Quantification of PINK1, p-PINK1 and NIX (n=6); F. Schematic diagram of TOM20 costaining with LC3, scale bar=20 μm (n=3); G. Schematic diagram of TOM20 costaining with LC3, scale bar=20 μm (n=3). GraphPad Prism 8 software was used to perform t tests for all the statistical analyses. All the values are the mean ± SEM. **p* < 0.05 indicates a statistically significant difference. HT22 cells transfected with empty plasmid were defined as the Vector group, and HT22 cells transfected with GSK-3β plasmid were defined as the GSK-3β-OE group.

**Fig.S3** **Effects of β-catenin overexpression and β-catenin inhibition on mitochondrial homeostasis.**

A. Statistical chart of cell viability (n=3); B. Representative protein bands that inhibit the regulation of LC3 by β-catenin; C. Quantification of LC3 (n=6); D. Representative protein bands for β-catenin overexpression validation; E. Quantification of β-catenin (n=6); F. TOM20 immunofluorescence, scale bar=20 μm; G. Statistical graph of the TOM20 immunofluorescence intensity (n=3); H. Schematic diagram of TOM20 costaining with LC3, scale bar=20 μm (n=3). GraphPad Prism 8 software was used to perform t tests for all the statistical analyses. All the values are the mean ± SEM. **p* < 0.05 indicates a statistically significant difference. HT22 cells transfected with empty plasmid were defined as the vector group, and HT22 cells transfected with β-catenin plasmid were defined as the β-catenin-OE group. HT22 cells treated with the solvent control were defined as the DMSO group, and HT22 cells treated with MSAB were defined as the MSAB group.

**Fig.S4** **Effects of HPC on mitophagic flux.**

A. Immunofluorescence co-localization staining of mitochondria, lysosomes and LC3II, scale bar=20 μm (n=3); B. Scatter plot and quantification of Pearson correlation coefficient (n=3). GraphPad Prism 8 software was used to perform t tests for all the statistical analyses. All the values are the mean ± SEM. **p* < 0.05 indicates a statistically significant difference.
